# Exome-wide evidence of compound heterozygous effects across common phenotypes in the UK Biobank

**DOI:** 10.1016/j.xgen.2024.100602

**Published:** 2024-06-28

**Authors:** Frederik H. Lassen, Samvida S. Venkatesh, Nikolas Baya, Barney Hill, Wei Zhou, Alex Bloemendal, Benjamin M. Neale, Benedikt M. Kessler, Nicola Whiffin, Cecilia M. Lindgren, Duncan S. Palmer

**Affiliations:** 1 Wellcome Trust Centre for Human Genetics, University of Oxford, Oxford, UK; 2 Big Data Institute, Li Ka Shing Centre for Health Information and Discovery, University of Oxford, Oxford, UK; 3 Target Discovery Institute, Centre for Medicines Discovery, Nuffield Department of Medicine, University of Oxford, Oxford, UK; 4 Program in Medical and Population Genetics, Broad Institute of MIT and Harvard, Cambridge, MA, USA; 5 Stanley Center for Psychiatric Research, Broad Institute of MIT and Harvard, Cambridge, MA, USA; 6 Analytical and Translational Genetics Unit, Department of Medicine, Massachusetts General Hospital, Boston, MA, USA; 7 Novo Nordisk Center for Genomic Mechanisms of Disease, Broad Institute of MIT and Harvard, Cambridge, MA, USA; 8 Data Sciences Platform, Broad Institute of MIT and Harvard, Cambridge, MA, USA; 9 Nuffield Department of Population Health, Medical Sciences Division, University of Oxford, Oxford, UK

**Keywords:** recessive, bi-allelic, compound heterozygosity, population genetics, phasing, longtudinal

## Abstract

The phenotypic impact of compound heterozygous (CH) variation has not been investigated at the population scale. We phased rare variants (MAF ∼0.001%) in the UK Biobank (UKBB) exome-sequencing data to characterize recessive effects in 175,587 individuals across 311 common diseases. A total of 6.5% of individuals carry putatively damaging CH variants, 90% of which are only identifiable upon phasing rare variants (MAF < 0.38%). We identify six recessive gene-trait associations (*p* < 1.68 × 10^−7^) after accounting for relatedness, polygenicity, nearby common variants, and rare variant burden. Of these, just one is discovered when considering homozygosity alone. Using longitudinal health records, we additionally identify and replicate a novel association between bi-allelic variation in *ATP2C2* and an earlier age at onset of chronic obstructive pulmonary disease (COPD) (*p* < 3.58 × 10^−8^). Genetic phase contributes to disease risk for gene-trait pairs: *ATP2C2*-COPD (*p* = 0.000238), *FLG*-asthma (*p* = 0.00205), and *USH2A*-visual impairment (*p* = 0.0084). We demonstrate the power of phasing large-scale genetic cohorts to discover phenome-wide consequences of compound heterozygosity.

## Highlights

• Biobank-scale genetic datasets can be phased and used to infer phenotypic consequences
• Incorporation of genetic phase can boost power in rare variant burden testing
• Bi-allelic variants in *ATP2C2* are associated with chronic obstructive pulmonary disease

## Graphical abstract

**Figure:**
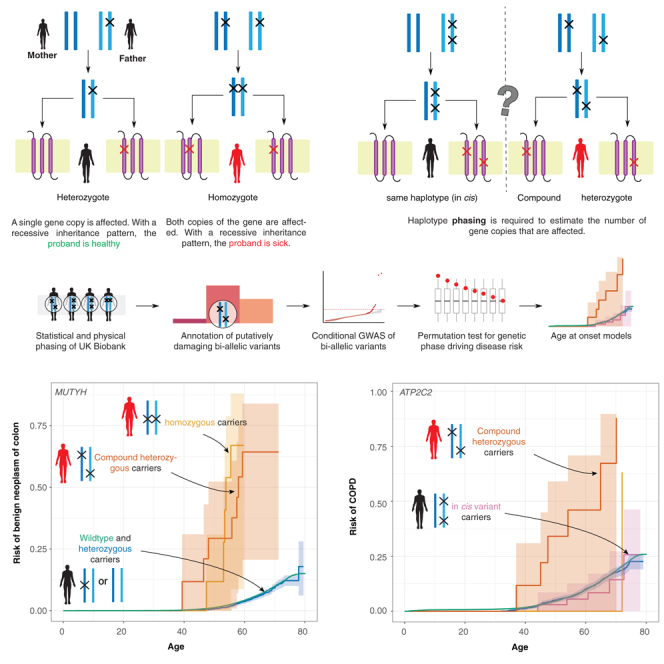

## Teaser

Lassen et al. investigate the role of specific rare genetic variants inherited from both parents in the development of common diseases. By applying statistical methods to analyze inheritance patterns, they link such mutations to diseases ranging from asthma to cancer in the UK Biobank.

## Introduction

Thousands of independent genetic variants have been robustly associated with common, complex human diseases, leading to important advancements in therapeutic development.^1^ Naturally occurring variants that disrupt protein-coding sequences are of interest in the context of drug discovery as they modulate potential biological targets, with measurable effects on human physiology.^2^^,^^3^ Thus, individuals who carry loss-of-function (LoF) variants on both the maternal and paternal copies of a gene are, in principle, experiments of nature, and their identification can help to determine causality between gene function and phenotype.^4^^,^^5^^,^^6^

Coding variants in a gene can either be homozygous, where both gene copies harbor the same variant, or compound heterozygous (CH), where both copies harbor different variants, usually at distinct genetic locations within the same gene locus. Alternatively, if two variants are located on a single gene copy, they are said to be in *cis*. Although both copies of a gene are disrupted in two-hit (CH or homozygous) carriers, analyses of the phenotypic impact of coding variation have typically ignored genetic phase information—that is, the separation or “phasing” of an individual’s genome into maternally and paternally derived alleles.^7^^,^^8^ Large-scale studies of bi-allelic damaging variation have generally been restricted to homozygotes in populations with excess homozygosity, such as Icelanders,^9^ Finns,^10^^,^^11^ and consanguineous populations.^12^ In contrast, CH is expected to be more common in outbred populations, but are understudied outside of rare disorders.^13^^,^^14^^,^^15^^,^^16^^,^^17^ While recent efforts have resulted in the characterization of bi-allelic variation in large-scale population cohorts, researchers have so far been unable to systematically link CH variants to disease.^18^^,^^19^

A series of methods exists to infer the genetic phase of two variants. “Phasing by transmission” employs family member genotyping and Mendelian inheritance principles,^20^ while “read-backed phasing” utilizes physical relationships among variants within sequencing reads.^21^ In large-scale biobanks, extensively genotyping family members is impractical, and short-read sequencing technologies only allow read-backed phasing for variants in close proximity. Therefore, “statistical phasing,” which models the generative process of newly arising genetic variation subject to recombination and mutation,^20^^,^^22^^,^^23^^,^^24^^,^^25^ is typically used to phase haplotypes in genetic biobank data. Obtaining high-quality statistically phased genetic data requires large sample sizes (10^5^–10^6^ individuals), and tends to require large reference panels.^23^ Furthermore, statistical phasing is more error prone for rare variants, which are precisely the collection of variants that we would like to investigate as they are *a priori* more likely to be deleterious variants of large effect under purifying selection. This difficulty in the accurate statistical phasing of rare variation has historically deterred the analysis of CH variants in biobanks. However, recent advances in statistical phasing,^19^ achieved by combining common variation across genotyping arrays and exome sequencing (ES) to create haplotype “scaffolds”^24^ enables accurate phasing of rare variants. By using this new accurate phase information, which extends down into rare allele frequencies, we can identify damaging CH variants to expand the pool of identifiable two-hit carriers and screen for phenotypic consequences.

We describe and apply a systematic analytical approach to test for autosomal bi-allelic effects, gene by gene, across up to 311 traits in the UK Biobank (UKBB) 200K ES release, combining both CH and homozygous variation. We iteratively refine candidate associations by adjusting for polygenic background, nearby common variant risk, and rare variant burden within the analyzed gene. Our approach identifies both known and novel bi-allelic-trait associations that we replicate using a subset of the UKBB 450K ES release, distinct from samples in the 200K ES release.

## Results

### Accurate phase inference and validation using parent-offspring trios and short-read sequences

We identified 13,377,336 high-quality variants in 176,935 individuals exome sequenced in the UKBB 200K release (STAR Methods). To identify variants co-occurring on the same haplotype (in *cis*) or on opposite haplotypes (in *trans*) gene by gene, we jointly phased ES and genotype array data in the UKBB using SHAPEIT5^26^ (STAR Methods) following an investigation into the performance of popular phasing software (Table S1). Rare variants (minor allele frequency [MAF] <0.001) are assigned a posterior probability (PP) of true haplotype assignment, known as the phasing confidence score. Confidence in our ability to accurately statistically phase variants decreases with MAF (Figure S1). However, we *a priori* expect a disproportionate recessive damaging signal to reside in CH variants with at least one rare variant, and as a result, choosing a PP cutoff represents a trade-off in the signal-to-noise ratio. Following phasing, we restricted our study to 176,935 individuals of genetically ascertained non-Finnish European (NFE) ancestry (STAR Methods).

**Figure 1:**
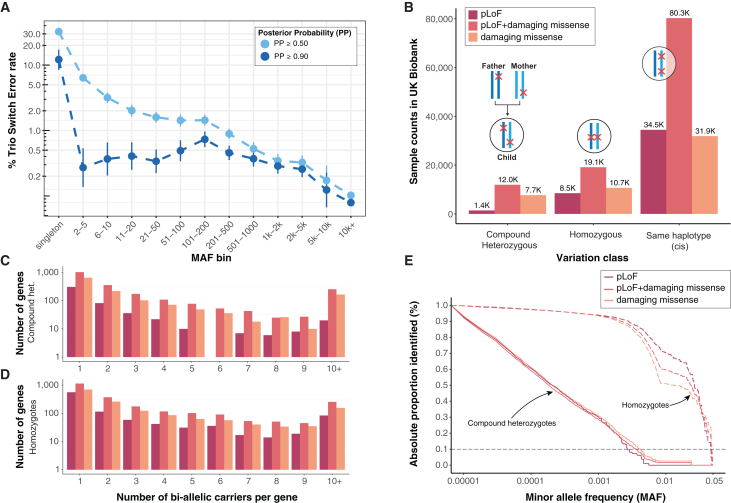
CH variants composed of at least 1 ultra-rare variant (MAC ≤ 10) can be robustly identified in large-scale biobanks (A) Trio SER depicted on the y axis as a function of MAC bin (x axis) for phased variants with MAF ≤ 5%, stratified by phasing confidence score PP ≥ 0.5 or PP ≥ 0.9. Error bars display 95% binomial confindence intervals. (B) Counts of samples harboring different classes of variation with at least 2 variants in UKBB. Each set of 3 bars depicts the number of individuals with at least 1 CH variant, homozygous variant, or multi-hit (*cis*) variant, respectively. Here, we define a CH pLoF + damaging missense variant as any combination of pLoF and/or damaging missense variation on opposite haplotypes. A qualifying carrier for each bar occurs according to the configuration displayed above the bars and is grouped by variant consequence according to the color legend. (C and D) Number of CH or homozygous carriers per gene. (E) One minus cumulative fraction (y axis) of homozygous (dashed line) and CH carriers as a function of lowest MAF (x axis) in bi-allelic variant pairs for which both variants phased at PP ≥ 0.9 (solid line), stratified by variant consequence according to the color key.

To assess statistical phasing quality, we benchmarked against phasing determined with parent-offspring trio data and performed read-backed phasing using short-read sequences. We first quantified phasing quality before and after filtering by PP ≥ 0.9 at the genotype level in 96 parent-offspring trios by calculating switch error rates (SERs), estimated using Mendelian transmission, across 2,044,234 unique variants stratified by minor allele count (MAC) (Figures 1A and S2; Tables S2 and S3). Across the 96 children, 93.1% of the protein coding genes contained variants that were phased without switch errors (Table S4). In SHAPEIT5, singletons are phased by identifying the longest shared identity by descent (IBD) segment between the two haplotypes in the target individual and those in the population. The minor allele is then assigned to the haplotype with the shortest shared IBD segment, as a short IBD segment indicates an older common ancestor providing more time for a germline variant to occur. Consistent with previously reported estimates in ES and genotyping array data,^19^ we observe a singleton SER of 32.4% (95% confidence interval [CI] = 30.2%–45.6%) prior to filtering by PP. Upon restricting to PP ≥ 0.9, SERs among singletons (MAC = 1) reduce to 12.1% (95% CI = 8.42–17.2). By comparison, rare variants with 2 ≤ MAC ≤ 5 exhibit SERs of 0.27% (95% CI 0.13–0.53) after applying the PP filter (Figure 1A).

Although the calculation of SER using trios is the gold standard approach for phasing quality estimation,^23^ it is limited by the number of parent-offspring trios available. For this reason, we also performed read-backed phasing of 453,514 unique pairs of variants using UKBB short-read sequences on autosomes in 49,756 NFE individuals using WhatsHap^27^ (STAR Methods). While read-backed phasing only permits ascertainment of genetic phase among pairs of variants spanning one or a few overlapping short-read sequences (with typical lengths of up to 250 bp), read-backed phasing accuracy is independent of allele frequency, and therefore represents an orthogonal approach to evaluating the quality of statistically phased variation. Consistent with trio-SER, we observed increasing agreement between pairs of statistically and read-backed phased variants with increasing MAC (Figure S3; Table S5). For singletons, we observed that with increased PP, phasing accuracy increased, while the total proportion of retained singletons decreased (Figure S4). Filtering to phased variants with PP ≥0.9 at the genotype level, singletons and variants with 2 ≤ MAC ≤ 5, agreement between read-backed phasing, and statistical phasing was 86.34% (95% CI = 85.68%–86.98%) and 99.37% (95% CI = 99.33%–99.41%), respectively (Figure S5; Table S5).

Taken together, our benchmarking suggests that statistical phasing of the UKBB dataset is of high quality for rare to ultra-rare variants and even singletons, increasing our confidence in the identification of damaging CH variation. Given our observations of well-calibrated PP and the distribution of phasing confidence binned by MAC, we selected the empirical cutoff of PP ≥0.9 at the genotype level to retain 6,352,396 variants (12% of which are singletons) for downstream characterization and testing (Table S2).

### Identification and examination of CH variation in the UKBB

To interrogate the functional role of mono- and bi-allelic variation in the population, we annotated 6,352,396 variants (762,843 of which were singletons) with PP ≥ 0.9 and MAF ≤ 5% across 17,998 autosomal protein-coding genes. We enriched our search for variants with putatively large effect sizes by restricting analyses to two categories of predicted damaging variation. First, we annotated 146,299 (22,380 singletons) high-confidence protein truncating variants, including stop-gain, essential splice, and frameshift variants identified as high confidence by LoF transcript effect estimator (LOFTEE),^28^ which we refer to as putative LoF (pLoF) variants. Second, we annotated 242,859 (32,199 singletons) missense variants classified as damaging by both Rare Exome Variant Ensemble Learner (REVEL) score ≥0.6 and Phred scaled Combined Annotation Dependent Depletion (CADD) score ≥20, or LOFTEE low confidence protein-truncating variants; we refer to these variants collectively as damaging missense/protein altering (Figure S6; Table S6). For each individual, we then determined the set of genes predicted to be affected by pLoFs + damaging missense/protein-altering variants in a CH, homozygous, or in *cis* state on the same haplotype.

As we *a priori* expected that essential genes would be less permissible to bi-allelic damaging variants when compared to non-essential genes, we investigated tolerance toward predicted bi-allelic pLoF and pLoF + damaging missense/protein-altering variants across the genome. As some genes carry bi-allelic variants more often than others (owing to a variety of factors such as gene length and baseline mutation frequency^29^), we fit counts of the number of individuals carrying bi-allelic variants per gene using a Poisson regression model, accounting for variation in gene length and mutation rate (STAR Methods; Tables S7 and S8). Both pLoF and pLoF + damaging missense/protein-altering bi-allelic variants (homozygous and CH) were significantly depleted in five of the six analyzed essential gene sets $\left(p<\frac{0.05}{6}\approx 0.0083\right)$ (Figure S7). Conversely, across three non-essential gene-sets, bi-allelic pLoFs + damaging missense/protein-altering variants were enriched among LoF-tolerant genes^29^ $\left(p\leq \frac{0.05}{3}\approx 0.0167\right)$. We found that the degree and direction of effects were consistent across CH, homozygous bi-allelic, and heterozygous variants (Figure S7) and concordant with previous study on CH depletion using the same gene set definitions.^19^ Additional gene set enrichment analyses are provided in Table S7.

In founder^9^ and bottle-necked^10^ populations, some alleles are enriched to high frequency by chance, resulting in better-powered association studies for the subset of variant alleles that are inherited from the parental population at higher frequency.^6^ To explore the diversity of bi-allelic variation in UKBB, a largely outbred population, we enumerated two-hit carriers across 176,587 individuals (Table S8). We observed complete bi-allelic knockout of 1,174 unique genes strictly due to pLoF variants, identifying 1,431 (0.8%) CH and 8,582 (4.8%) homozygous individuals with bi-allelic pLoF variants in at least one gene (Figure 1B). Across genes, 307 (26.1%) CH and 560 (47.7%) homozygous knockouts were observed only in a single individual (Figures 1C and 1D). Our simulations (STAR Methods) closely recapitulated the empirically observed patterns of bi-allelic pLoF events, with strong correlation between simulated and observed homozygous (*R* = 0.996, *p* < 2.2 × 10^−16^) and CH (*R* = 0.932, *p* < 2.2 × 10^−16^) pLoF events (Figure S8). We reasoned that the inclusion of damaging missense/protein-altering variants in addition to pLoFs would expand the number of identifiable damaging bi-allelic variants compared to assessing the two categories independently. Across 3,288 unique genes, we observed 11,491 (6.5%) CH and 17,863 (10.1%) homozygous carriers of pLoF + damaging missense/protein-altering variants. Of these, 1,112 (0.6%) CH and 436 (0.2%) homozygotes were carriers of bi-allelic pLoF + damaging missense/protein-altering variants in genes linked to traits with an autosomal recessive mode of inheritance in OMIM.^30^ Consistent with previous observations,^18^ we generally observed a higher prevalence of carriers with variants in *cis* compared to CH, with over one-third of individuals (64,555, 36.6%) carrying ⩾2 pLoF + damaging missense/protein-altering variants on a single haplotype (Figure 1B).

To better understand the evolutionary dynamics giving rise to pathogenic variants in *trans*, we examined the spectrum of allele frequencies of the constituent variants among our confidently called damaging CH variants. CHs variants tend to comprise two variants where one resides on a common haplotype, while the other resides on a rare haplotype, with a median difference in MAC of 1,181 (Figures S9 and S10). Approximately 90% of CH-constituent variants have MAF ≤ 0.0038, compared to homozygotes in which 90% are detected at MAF ≥ 0.0022 (Figure 1E), suggesting that identifying deleterious bi-allelic CH variants requires reliable phasing of rare alleles (Figures S11 and S12).

Multiple studies have assessed the prospects of ascertaining bi-allelic LoF variation at larger sample sizes in consanguineous, bottle-necked, and outbred populations.^6^^,^^12^ To investigate empirically how the number of unique genes with bi-allelic variants scales in an outbred population, we performed down-sampling of UKBB participants. Consistent with the previous literature, additional CH and homozygous variants can be inferred by considering both pLoF and damaging missense/protein-altering variation at even larger sample sizes (Figure S13).

### Systematic evaluation of bi-allelic effects on common disease

We performed a series of association analyses using the scalable and accurate implementation of generalized mixed model (SAIGE),^31^ a generalized mixed-model association testing framework that uses a saddle point approximation to provide accurate *p* values for traits with extreme case-control ratio imbalance. This allowed us to investigate the effects of bi-allelic variants in 176,587 individuals across 311 phenotypes with varying population prevalence identified from secondary care electronic health records (STAR Methods). We restricted to 952 protein-coding genes with at least 5 individuals carrying bi-allelic variants in the same gene, which allowed us to detect odds ratios (ORs) ≥10, for traits at approximately 2% population prevalence, with 80% power at exome-wide significance (Bonferroni $p<\frac{0.05}{952\times 311}\approx 1.68\times 10^{-7}$) (STAR Methods; Figure S14). Using simulations, we confirmed our ability to detect recessive signals of association with well-calibrated false positive rates across a range of effect sizes (STAR Methods; Figures S15A–S15C). We tested a total of 299,854 gene-trait combinations and identified 7 gene-trait associations following stringent Bonferroni correction (*p* < 1.68 × 10^−7^) (Figure S16; Table S9). We observed that excluding singletons had a minimal impact on the resulting gene-trait associations (Figure S17), likely due to the limited number of CH variants comprising at least one singleton (Figure S18). Finally, we performed confirmatory replication analysis in the remainder of the UKBB (STAR Methods) and found that 7 of 7 (100%) gene-trait associations with at least 5 bi-allelic carriers replicated at *p* < 0.05 (Table S10).

A recessive gene-trait association may be influenced by a variety of genetic factors unrelated to CH or homozygous status, such as polygenic background or through genetic tagging of a nearby common variant association. To mitigate these factors, we created a pipeline to condition on external genetic effects within the gene-trait regression model (STAR Methods). We trained polygenic risk scores (PRSs) for 111 significantly heritable traits ($h_{\text{snp}}^{2}\;p<0.05$ and *n*_eff_ ⩾ 5,000) using LDPred2^32^ (STAR Methods; Table S11), a tool that allows PRS derivation based on summary statistics and linkage information. To control for polygenic risk and potentially boost power for association,^33^ we included the off-chromosome PRS as an additional covariate (Table S9). We observed that controlling for PRS had no significant influence on the binary case-control association testing, and the resulting *p* values were altered by less than a single order of magnitude with the incorporation of PRS (Figure S19). To capture the effects of any causal common variants in linkage disequilibrium with the pLoF or damaging missense/protein-altering variants constituting the CH or homozygous variant, we further conditioned on nearby (within 1 Mb of the associated gene) common (MAF ≥ 1) variant association signals (STAR Methods; Table S12), which abrogated (*p* > 0.05) the signal of a single gene-trait pair.

Lastly, we investigated whether any of the identified putative recessive associations could be accounted for by assuming an additive genetic architecture. To do this, we counted the number of gene copies affected by pLoF + damaging missense/protein altering variants in each individual. For each putative recessive association, we re-ran the analysis while simultaneously conditioning on the number of affected haplotypes. We also employed a complementary variant-level approach and repeated the analysis, conditioned on all low-frequency (MAC >10, MAF ≤ 5) and ultra-rare (MAC ≤ 10) damaging variants (pLoF + damaging missense/protein-altering), including those that constitute the bi-allelic variant in question. Among the remaining six Bonferroni significant associations, none of the associations were abrogated after conditioning on additive effects (Table S9).

**Figure 2:**
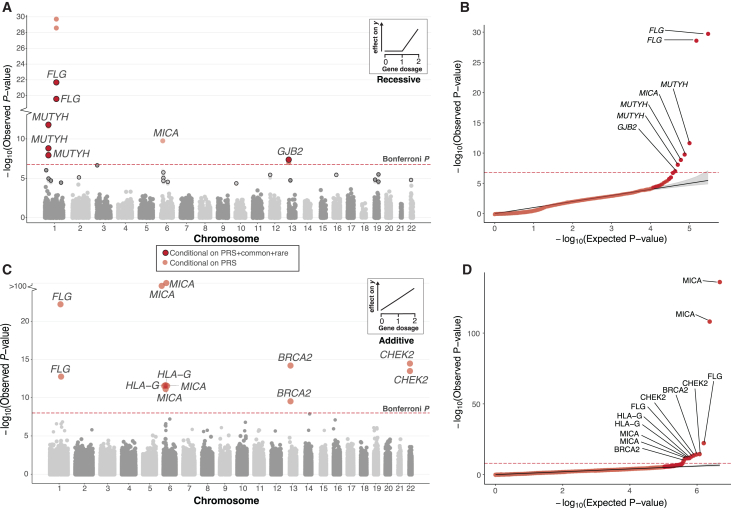
Conditional recessive and additive modeling of gene copy disruption in 311 phenotypes across 176,587 participants (A) Recessive Manhattan plot depicting log_10_-transformed gene-trait association *p* values against chromosomal location. Associations are colored red if they are Bonferroni (*p* < 1.68 × 10^−7^) significant. Transparent coloring represents the resulting *p* value when conditioning only on PRS, whereas solid coloring with black outline represents the *p* value derived after conditioning on off-chromosome PRS, nearby (500 kb) common variant association signal, and rare variants within the gene when applicable (STAR Methods). The Bonferroni significance threshold is also displayed as a red dashed line. A gene may appear multiple times if it is associated with >1 phenotype. A qualifying example of the recessive inheritance pattern is shown at the top right of the panel: disruption of both gene copies results in an effect on the phenotype. (B) Quantile-quantile (Q-Q) plot for genes with bi-allelic damaging variants after conditioning on off-chromosome PRS. The shaded area depicts the 95% CI under the null. Gene-trait associations passing Bonferroni significance are labeled accordingly. (C and D) Additive Manhattan plot and corresponding Q-Q plot for genes with mono- and bi-allelic damaging variants associated with at least 1 phenotype after conditioning on off-chromosome PRS when applicable (STAR Methods). No additional conditioning was performed in this analysis. Gene-trait associations are colored red if they are Bonferroni (*p* < 9.8 × 10^−9^) significant. The additive inheritance model is depicted at the top right of the panel; each affected haplotype results in a incremental effect on the phenotype.

Together, these analyses refined the list of putative gene-trait associations to six Bonferroni associations after stringent Bonferroni correction and conditioning (Figures 2A and 2B; Table S9) comprising three unique genes and six traits. Notably, only three of the six associations remained significant (Bonferroni *p* < 1.68 × 10^−7^) when restricted to only CH variant carriers, and just one of six when testing homozygous variants alone, underscoring the power of jointly analyzing these variant sets (Figure S20).

We observed recessive gene-trait relationships across multiple physiological domains (respiratory, neoplasm, skin, ear, and mastoid). All six associations that met the significance threshold after Bonferroni correction and conditioning (*p* < 1.68 × 10^−7^) have been reported previously in the literature. For example, individuals with bi-allelic variants in *MUTYH*, a gene involved in oxidative DNA damage repair,^34^ are at significantly increased risk of developing colorectal cancer (log_10_(OR) = 4.7 [95% CI = 3.38–6.01], *p* = 2.2 × 10^−12^). We also find that bi-allelic variants in *FLG* increase the risk of both asthma^35^ (log_10_(OR) = 3.3 [0.26–0.39], *p* = 2.09 × 10^−22^) and dermatitis^36^ (log_10_(OR) = 0.28 [0.22–0.33], *p* = 2.65 × 10^−20^). In addition, we observe that bi-allelic variants in *GJB2* increase the risk of hearing loss^30^ (log_10_(OR) = 1.66 [1.05–2.26], *p* = 9.93 × 10^−8^).

**Figure 3:**
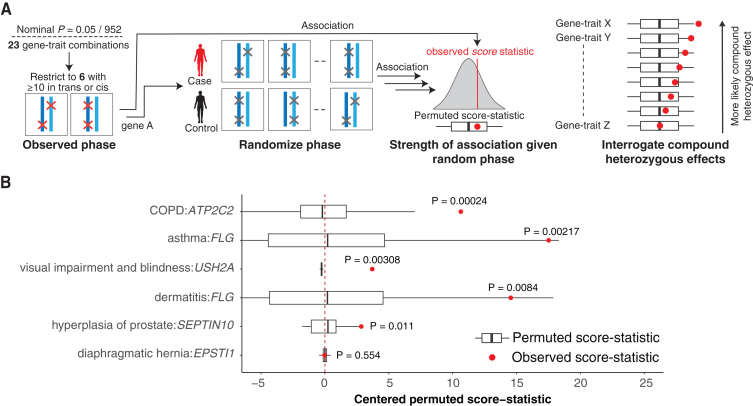
*In silico* permutation of genetic phase provides evidence for CH-specific effects (A) Overview of the permutation pipeline. To be sufficiently powered to detect effects, we considered 5 significant (*p* < 0.01) gene-trait pairs from the genome-wide analysis that have at least 10 individuals harboring pLoF or damaging missense/protein-altering variants on the same haplotypes or CH carriers. Then, we shuffled CH *trans* and *cis* labels across samples and re-ran the association analysis, resulting in a null distribution of permuted score statistics corresponding to the association strength in the absence of phase information. We derive the 1-tailed empirical *p* value by comparing the observed score statistics with the empirical null distribution. (B) The resulting distributions of permuted (white and black boxplots) and observed score statistic (red dot) for each gene-trait and the resulting empirical *p* value. *p* values shown in bold indicate Bonferroni significance ((*p* < 0.05/06 = 0.0083). Box and whisker plots display the quartiles of the empirical null distribution.

To assess the degree to which compound heterozygosity, rather than co-occurring variants on the same haplotype, drives disease risk, we permuted the genetic phase of observed pLoF + damaging missense/protein-altering variants within a gene to generate an empirical distribution of score statistics corresponding to disease-association strength in the absence of phase information (Figures 3A and 3B). To ensure a sufficiently large sampling distribution, we restricted our analysis to six nominally significant $\left(p<\frac{0.05}{952}\approx 5.25\times 10^{-5}\right)$ gene-trait combinations with at least 10 individuals who are either CH variant carriers or with ≥2 pLoF or damaging missense/protein-altering variants on the same haplotype (STAR Methods). We found evidence that the incorporation of CH variation significantly (Bonferroni *p* = 0.05/6 = 0.0083) increased the score statistic in three of the six analyzed gene-trait combinations. CH variants in *ATP2C2* are associated with an increased risk of chronic obstructive pulmonary disease (COPD) (*p* = 0.000238), and CH variants in *FLG* are associated with an increased risk of asthma (*p* = 0.002174), while CH variants in *USH2A* are associated with an increased risk of visual impairment and blindness (*p* = 0.000307) (Figure 3B). We identified two additional gene-trait association at nominal significance (*p* < 0.05): CH variants in *FLG* that are associated with an increased risk of dermatitis (*p* = 0.0092), and CH variants in *SEPTIN10* that are associated with hyperplasia of prostate (*p* = 0.011). Of these, *FLG*-asthma, *FLG*-dermatitis, and *USH2A*-visual impairment associations have previously been linked to disease in the CH state.^37^^,^^38^^,^^39^ These observations demonstrate on a large scale the effect of compound heterozygosity in driving disease susceptibility and, by extension, how appropriately integrating genetic phase can lead to increased power to discover gene-trait associations.

### Incorporation of genetic phase boosts power in gene-level rare variant burden models

Complementary to the recessive models described above, rare variant burden testing, which involves the aggregation of rare variants within a gene, has proven to be a robust method to collectively assess the phenotypic impact of rare variation across individuals. Rare variants are aggregated due to their low allele frequency leading to lack of statistical power for the detection of single-marker associations. However, these frameworks generally ignore the genetic phase within each individual, and therefore do not differentiate between scenarios in which multiple damaging variants reside on the same (in *cis*) or opposite (in *trans*) haplotypes, despite these two forms having potentially distinct functional and phenotypic effects. We conducted additive genome-wide association analyses by testing for associations between the number of disrupted gene copies (across 16,363 protein-coding genes with at least 10 haplotypes carrying pLoF + damaging missense/protein altering variation) on an individual and case status (across 311 phenotypes) (Figures 2C and 2D; STAR Methods). After adjusting for polygenic contribution, we found 12 significant associations after stringent multiple-testing correction (Bonferroni $p<\frac{0.05}{16,363\times 311}\approx 9.8\times 10^{-9}$; Table S13). Among the significant hits are previously reported associations, including association between the number of putatively damaged copies of *BRCA2* (*p* = 6.16 × 10^−15^) and *CHEK2* (*p* = 3.34 × 10^−15^) and breast cancer. We then compared our approach to an established gene-based method for rare variant burden testing, specifically SAIGE-GENE+.^40^ We applied SAIGE-GENE+ to the same variant set used in our study, comparing the *p* values derived from this method with those from our haplotype-collapsing approach (Table S14). This initial comparison revealed 5 of 12 (42%) associations with insufficient evidence to reach Bonferroni significance (*p* < 9.8 × 10^−9^) using SAIGE-GENE+ burden testing. Moreover, among our significant additive haplotype-based gene associations, 11 of 12 (92%) were more strongly associated than in SAIGE-GENE+ burden testing (one-sided sign test, *p* = 0.00317). Recognizing that our initial analysis deviated from the regular usage of SAIGE-GENE+, which ignores phasing confidence, we also performed an analysis without filtering variants by PP. This allowed us to increase the pool of variants and compare the performance of the haplotype-collapsing method with the standard application of the burden test. We observed that while SAIGE-GENE+ now recapitulated all 12 associations at the Bonferroni cutoff (*p* < 9.8 × 10^−9^), we found that 12 of 12 (100%) gene-trait associations were more strongly associated using the haplotype-based collapsing method (one-sided sign test, *p* = 0.00024).

### Non-additive effects of CH variants elevate lifetime risk of disease

**Figure 4:**
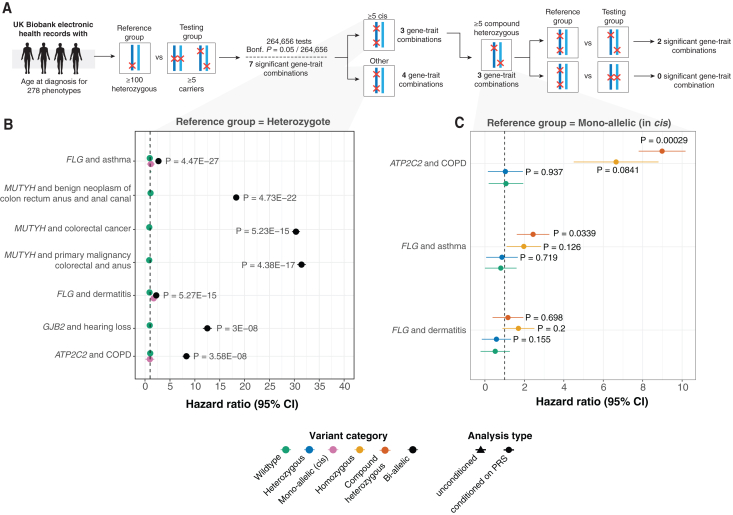
Age at diagnosis modeling reveals novel recessive effects driven by damaging bi-allelic variants (A) Flow diagram of our approach. To investigate whether homozygous and/or CH effects are associated with a difference in lifetime risk of disease development, we performed Cox proportional hazards modeling for gene-trait combinations in which ≥5 samples are 2-hit carriers (CH or homozygotes) and ≥100 samples that are heterozygotes. Among Bonferroni (Bonf.) significant associations (*p* < 1.89 × 10^−7^), we filter to gene-trait pairs for which at least 5 samples carry multiple variants disrupting the same haplotype and test for an association between CH or homozygous carrier status and lifetime disease risk (corresponding to HRs ≥1). (B) HRs when comparing CH and homozygous status versus heterozygous carrier status. Throughout, we display hazard ratios and corresponding *p* values after taking the polygenic contribution into account by conditioning on off-chromosome PRSs for heritable traits that pass our quality control cutoffs. *p* values following inclusion of polygenic contribution to disease status are provided where PRSs are predictive. HRs for gene-traits with ≥2 individuals with multiple *cis* variants on the same haplotype are displayed in pink. Only associations that pass the stringent Bonferroni significance threshold (*p* < 1.89 × 10^−7^) cutoff are illustrated. (C) HRs when comparing wild-type, heterozygous, CH, and homozygous carrier status against individuals that harbor ≥2 putatively damaging variants on the same haplotype. 95% CIs are shown in the figure.

Bi-allelic effects may be associated with earlier age at onset of disease, which is also often correlated with disease severity. We therefore explored whether CH and homozygous variants had longitudinal effects by evaluating age at diagnosis of 278 phenotypes with Cox proportional hazards models. To identify effects due to the disruption of both gene copies, as opposed to haploinsufficiency, we compared bi-allelic variant carriers against a reference group comprising carriers of a single heterozygous variant for each gene. We tested 267,400 gene-trait combinations with at least 5 bi-allelic variants (homozygotes or CH) and 100 heterozygotes (Figure 4A). After adjusting for polygenic risk via off-chromosome PRS, we identified seven gene-trait associations with significantly earlier age at diagnosis in bi-allelic variants compared to heterozygous carriers of pLoF + damaging missense/protein-altering variants (Bonferroni $p<\frac{0.05}{952\times 278}\approx 1.89\times 10^{-7}$; Figures 4B, 4C, and S21–S23; Tables S15 and S16). For six out of the seven Bonferroni significant gene-trait combinations, we found no evidence (p>0.05/7≈0.00833) that carrying a single heterozygous variant altered lifetime disease risk compared to carrying two copies of the reference allele. We also performed confirmatory replication analyses for 7 associations with at least 5 bi-allelic and 100 heterozygous carriers in the replication cohort (STAR Methods). We found that all 7 (100%) Bonferroni significant gene-trait associations replicated at (*p* < 0.05), including *ATP2C2*-COPD (replication *p* = 0.013), which has not previously been reported in the literature (Table S17).

We further sought to disentangle the effects of homozygous and CH variants on lifetime disease risk from that attributable to multiple damaging rare variant effects on a single haplotype. To do this, we analyzed these effects in the three gene-trait pairs with both (1) at least 5 CH and/or homozygous variants and (2) at least 5 individuals harboring ⩾2 variants on the same haplotype (Figure 4C; Table S18). Compared to individuals with a single disrupted haplotype, both homozygous and CH carriers of pLoF + damaging missense/protein-altering variants in *ATP2C2* were at an increased lifetime risk of developing COPD (homozygote hazard ratio [HR] = 6.65 [95% CI = 4.5–8.8], *p* = 0.084, CH HR = 8.98 [95% CI = 7.79–10.17]; *p* = 0.00028). Similarly, both homozygous and CH variants of *FLG* were at an increased lifetime risk of asthma (homozygote HR = 1.97 [95% CI = 1.1–2.84], *p* = 0.126, CH HR = 2.44 [95% CI = 1.61–3.26], *p* = 0.033) and dermatitis (homozygote HR = 1.7 [95% CI = 0.88–2.5], *p* = 0.20, CH HR = 1.16 [95% CI = 0.38–1.94], *p* = 0.7) (Figure 4C). For these gene-trait relationships, mono-allelic carriers have no increase in risk of disease compared to wild types, whereas bi-allelic carriers have a significant increase in lifetime risk of disease.

### Biological insights into common complex disorders implicated by CH variation

**Figure 5:**
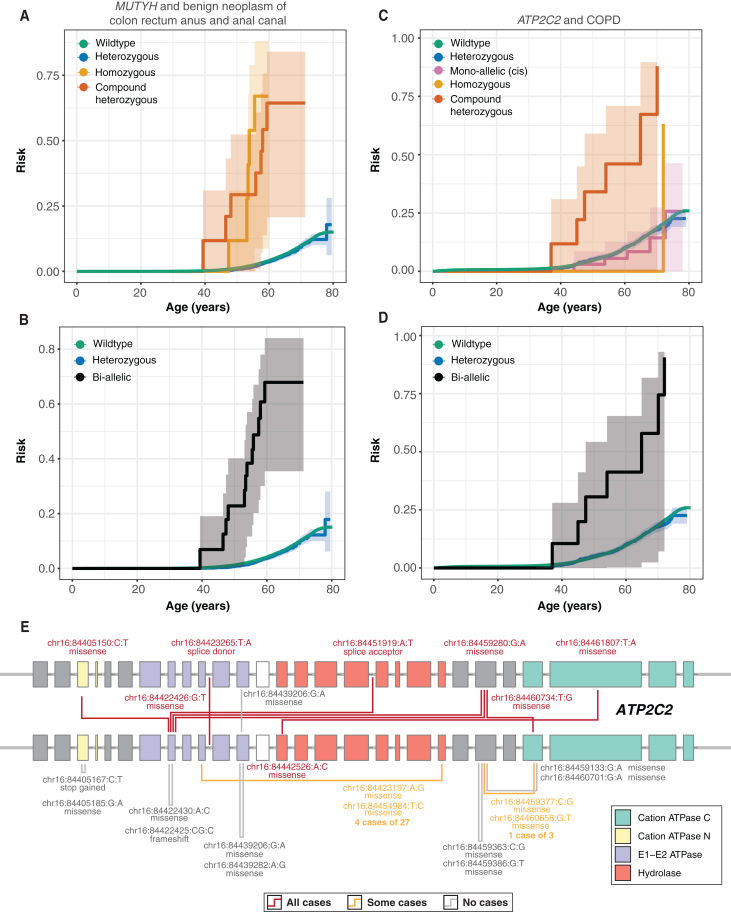
Trajectories of haplotype disruption in common diseases (A and B) Kaplan-Meier survival curves for CH (red), homozygous (orange), heterozygous carriers (blue), and single disruption of haplotypes (pink) due to pLoF or damaging missense/protein-altering mutations. Shaded regions indicate 95% confidence intervals for risk estimates. Wild types and bi-allelic variants (CH or homozygous) are shown with green and black lines, respectively. Both CH and homozygous *MUTYH*-variant carriers are at elevated lifetime risk of developing benign neoplasm of the colon compared to heterozygous carriers and wild types. (C and D) Kaplan-Meier survival curves for *ATP2C2* mono- and bi-allelic variant carriers. Carriers of CH variants develop COPD earlier compared to heterozygotes carriers and wild types. Moreover, individuals who harbor a single putatively disrupted haplotype due to ≥2 damaging variants develop COPD at the same frequency as heterozygotes and wild types. (E) Gene plots for *ATP2C2*, displaying protein coding variants for samples that carry ≥2 pLoF or damaging missense/protein-altering variants stratified by exon or intron. CH variants, multiple variants in *cis*, and homozygous variants are highlighted by lines joining the positions of co-occurring variants in a sample. Lines are colored by the number of cases for the shown variant configurations, with gray lines indicating no observed samples are cases, orange lines indicating some some samples are cases, and red lines indicating that all observed samples are cases. Variants are labeled by position (GRCh38) and according to inferred consequence (missense, stop gain, splice acceptor/donor). Protein domains are highlighted accordingly.^41^

Six of the seven gene-trait combinations for which we identify Bonferroni significant associations with lifetime disease risk are also significant in our cross-sectional recessive association analysis (Table S19). The six have previously been reported in the literature, albeit without age at onset effects. These include *MUTYH* and colorectal cancer, *GJB2* and hearing loss, and a pleiotropic association of *FLG* with both dermatitis and asthma (Figures 5A, 5B,^41^ and S22). We find that *ATP2C2*-COPD is a novel candidate association (*p* = 3.58 × 10^−8^) with plausible mechanistic effects. All constituent variants and allele frequencies for Bonferroni significant associations are provided (Table S20).

*ATP2C2*, a calcium-transporting ATPase linked to surfactant protein D levels via an intronic variant (rs9927461, a causal risk factor for COPD),^42^ is associated with COPD in our gene-trait analyses (HR = 8.3 [95% CI = 7.54–9.05], (*p* < 3.56 × 10^−8^). As we did not observe any nearby (1 Mb upstream or downstream) common variants in *ATP2C2* associated with cross-sectional COPD (all *p* > 5 × 10^−6^), the association between bi-allelic variants of *ATP2C2* and COPD potentially is driven by the unique configurations of CH damaging missense (*n* = 7) and pLoF (*n* = 1) variants that primarily reside in functional protein domains (Figures 5E and S24; Table S21). Seven of the eight (87.5%) individuals we identified as bi-allelic carriers of damaging variation in *ATP2C2* (6 CH and 2 homozygous) were diagnosed with COPD (median age of diagnosis = 54.1 [interquartile range = 46.2–67.5] years) (Figures 5C and 5D). In contrast, only 6.9% of individuals harboring multiple pLoF + damaging missense/protein-altering variants on the same *ATP2C2* haplotype were diagnosed with COPD, with the same median age of diagnosis (60.8 [53.7–67.9] years) as both heterozygote carriers (58.0 [48.5–64.1] years) and those with wild type (59.2 [51.3–65.1] years).

*FLG* plays a pivotal role in the differentiation and maintenance of skin barriers.^35^ *FLG* variants have been selectively associated with individuals with both asthma and atopic dermatitis, but not with those who have asthma without atopic dermatitis.^36^ Our findings indicate that individuals carrying a single deleterious *FLG* allele face increased risk of dermatitis (HR = 1.11 [95% CI = 1.05–1.19], $p\approx 7.2\times 10^{-5}$), but not asthma (HR = 1.06 [95% CI = 1.01–0.12], *p* = 0.018), when compared to wild types. In contrast, individuals carrying two variant alleles have an increased risk of developing both dermatitis (HR = 2.23 [95% CI = 2.03–2.43], *p* = 5.27 × 10^−15^) and asthma (HR = 2.68 [95% CI = 2.51–2.87], *p* = 4.47 × 10^−27^), suggesting a recessive mode of inheritance for *FLG*-related asthma and a semi-dominant inheritance mode for *FLG*-related dermatitis.^30^ This implies that the loss of a single *FLG* copy can result in dermatitis, while the loss of both copies can lead to asthma. Together, this may help clarify why *FLG*-related asthma is seldom observed without the presence of *FLG*-related dermatitis.

## Discussion

In this large biobank-scale effort, we systematically interrogated the role of bi-allelic coding variants in genes conferring risk for common complex diseases. In the cross-sectional and longitudinal recessive analysis, we identified six and seven significant gene-trait associations after stringent Bonferroni correction, respectively. In the cross-sectional analysis, only 2 of the 6 (33%) associations we identified would have been discovered considering homozygotes alone, whereas in the longitudinal analysis 5 of the 7 (71%) associations would have been discovered without considering compound heterozygosity. Collectively, the associations both replicate established relationships in the literature and identify a previously unreported gene-trait association for binary phenotypes across the common disease spectrum.

We show that the 90% of deleterious CH variants occur at MAF < 0.38%. Given that phasing quality is directly correlated with allele frequency, it is essential to filter to the set of variants phased at high confidence to eliminate false positive identifications. Here, we quantified the increase in phasing quality using Mendelian inheritance logic in parent-offspring relationships and compared pairs of statistically phased variants to read-backed phased variants using short-read sequences. While read-backed phasing is computationally expensive and restricted to variants in close proximity, we demonstrate that it can be employed to evaluate statistical phasing quality in cohorts that lack trio relationships, with error rates comparable to those of trio SERs.

CH disease associations have mainly been explored in rare disorders,^13^^,^^14^^,^^15^^,^^16^^,^^17^ but they seldom have been investigated in the study of common disease. This is due to the low prevalence of variants in the CH state and the genetic architecture of common complex traits, which are typically influenced by environmental factors and numerous loci with low to modest contribution to risk. In this study, we addressed these challenges and offered multiple lines of evidence to demonstrate the role of CH effects in driving disease risk for common traits. We employed two complementary analyses to detect gene-trait associations: a genome-wide logistic association analysis and a time-to-event model. Through these methods, we identified associations in which variants in the homozygous or CH state resulted in increased disease risk compared to wild types and individuals carrying multiple pathogenic variants on the same haplotype. Our findings show that for certain gene-trait pairs, individuals with a single disrupted gene copy have a risk of developing disease that is virtually indistinguishable from that of wild types, suggesting non-additive gene dosage effects. Furthermore, by permuting the genetic phase, we found evidence that the incorporation of confidently phased CH variants can boost power to detect associations in common disease. Collectively, our results emphasize the importance of considering each individual’s specific genetic context when assessing their genetic risk in a clinical setting. Simply identifying the presence of multiple pathogenic variants in a gene, disregarding the phase, may not be sufficient to fully understand an individual’s risk profile.

Many common complex traits have polygenic architectures, which should be accounted for when performing gene-trait association testing. The presence of bi-allelic variants in individuals with such diseases might be coincidental and not causally related to the trait, which may instead be a result of a high polygenic risk. However, across the significant recessive genome-wide associations, we observed that the inclusion of PRS as a covariate affected the resulting association *p* value by less than an order of magnitude for the binary traits we analyzed. While we were only able to account for the polygenic contribution to disease development for 111 diseases with significant common variant heritability in the UKBB, due to low case numbers, these observations suggest that the incorporation of polygenic background has limited influence on the degree of association when evaluating ultra-rare variation across binary traits.

We found that the majority of bi-allelic gene-disease associations are driven by variant combinations containing at least one missense variant, which would have been excluded under a stricter high-confidence pLoF criterion. Although our less stringent inclusion threshold enabled us to identify a greater number of bi-allelic variants, it is likely that some damaging missense or protein-altering variants would incorrectly be predicted as damaging, or they may exhibit gain-of-function rather than LoF effects, consequently reducing the signal-to-noise ratio in our analyses. Even knockouts by bona fide pLoF variants may result in only partial gene inactivation, and not necessarily complete gene knockdown. Additionally, pLoF variants may be “rescued” and not lead to complete or even partial LoF. While we show that including damaging missense/protein-altering variants to define bi-allelic variants can improve power for certain phenotypic associations, further manual curation and experimental validation will be required to demonstrate that these variants truly result in LoF.

The likelihood of damaging alleles occurring on the same haplotype is influenced by a complex interplay of factors, including population structure and balance between selection, drift, mutation, and recombination. We and others^18^ have found that damaging CH variants occur less frequently than multiple damaging variants affecting the same haplotype, suggesting that in certain circumstances, natural selection operates on a haplotype level. Once an LoF variant occurs and expands in the population, the affected haplotype has no selection against additional acquisition of damaging mutations. This has implications for association studies investigating CH effects by counting the number of damaging variants in a gene while attributing equal probability to each of affecting each haplotype,^8^ as such frameworks may overestimate the frequency of CH events.

“Human knockouts” have been extensively discussed in the context of therapeutic development. Examining both bi- and mono-allelic carriers can help one assess the safety of therapeutic interventions by analyzing how varying degrees of target modulation affect biological response.^3^^,^^6^ We showcased several gene-trait relationships where the number of affected haplotypes influences the lifetime risk of disease, potentially representing the manifestation of adverse events, which are important endpoints in clinical trials. The absence of adverse events in mono-allelic carriers can imply that partial pharmacological inhibition of a target may be a safe and effective approach. However, adverse effects observed in bi-allelic carriers of damaging variation within the same locus could indicate potential risks associated with complete target inhibition. A natural extension of this work could involve investigating mono- and bi-allelic effects on quantitative outcomes, such as serum proteins. Changes in biomarkers (or other continuous outcomes) may reflect direct or indirect consequences of gene modulation and could serve as potential pharmacodynamic biomarkers commonly used to assess target engagement in clinical trials.

This work showcases the value of statistical phasing of damaging rare variants and that association analyses that account for compound heterozygosity can be better powered for gene-trait discovery. We showed that this approach can be employed to discover well-established and novel non-additive and additive gene-trait relationships across a wide range of disease etiologies. From a clinical perspective, we demonstrated the importance of interrogating the genetic phase when dealing with CH variants in traits with recessive modes of inheritance. This is an important step toward uncovering the phenome-wide consequences of bi-allelic disruption across the human genome.

### Limitations of the study

We acknowledge several limitations to our study. First, the accuracy of statistical phasing decreases with the MAF. Our study encompasses ultra-rare variation (MAF < 0.001%), which we filtered to confidently phased heterozygous variants (PP < 0.9). This criterion excludes variants that could form CH relationships, potentially leading to an underestimation of CH events, especially among those that constitute rare variants and singletons. Second, we applied a conservative threshold for statistical significance (p < 1.68 × 10^−^^7^) allowing us to replicate all associations. However, caution should be exercised when interpreting findings that are less significant than this Bonferroni significance threshold, especially those involving a low frequency of bi-allelic variants and low case counts. These conditions can result in instability and lower power in the mixed-model framework, particularly for rare binary traits.^43^ Third, this work is based on coding mutations within protein-coding regions. Including splicing variants and non-coding variants, such as those in untranslated regions, could enhance the power of the analyses and potentially lead to further associations.

Lastly, our study was conducted within the UKBB using individuals of European ancestry. It is possible that the number of CH events and their prevalence across the genome may vary across non-European populations. Other population structures could affect the power for association. For example, in populations such as the Finns, where bottlenecks have caused rare variants from founders to rise to appreciable frequencies, there could be increased detection power for those alleles.^10^ Similarly, in consanguineous populations, the prevalence of homozygous pLoF genotypes increases due to parental relatedness. This increases the number of identifiable bi-allelic genotypes leading to a potential increase in power for assocation analysis. This is particularly relevant for members of the East London Genes & Health cohort^44^ or other cohorts with participants reporting high degrees of consanguinity. It will be critical to apply similar approaches in other cohorts encompassing diverse populations to further explore this diversity.

## STAR★Methods

### Key resources table

**Table:** 

REAGENT or RESOURCE	SOURCE	IDENTIFIER
**Deposited data**		

UK Biobank Exome and Genotype data	Bycroft et al.^45^	https://www.ukbiobank.ac.uk/
Summary statistics	This paper	https://doi.org/10.5281/zenodo.10939698

**Software and algorithms**		

SAIGE (1.1.9)	Zhou et al.^40^	https://www.nature.com/articles/s41588-020-0621-6
HAIL (0.2.97)	Hail team^44^	https://github.com/hail-is/hail
SHAPEIT5 (5.0.0)	Delaneau et al.^26^	https://github.com/odelaneau/shapeit5
LDpred2 (1.12.1)	Prive et al.^32^	https://github.com/privefl/bigsnpr
PLINK (1.9)	Chang et al.^41^	https://www.cog-genomics.org/plink/1.9/
BCFtools (1.12)	Danecek et al.^46^	https://samtools.github.io/bcftools/
Whatshap (1.6)	Martin et al.^27^	https://whatshap.readthedocs.io/en/latest/
Variant Effect Predictor (95)	McLaren et al.^47^	https://www.ensembl.org/info/docs/tools/vep/index.html
Gene lists	Multiple authors^28^^,^^48^^,^^49^^,^^50^^,^^51^^,^^52^	https://github.com/macarthur-lab/gene_lists
Main pipeline code	This paper	https://github.com/frhl/wes_ko_ukbb_nexus,10.5281/zenodo.10926001

### Resource availability

#### Lead contact

For additional information, as well as requests regarding resources and reagents, please direct your inquiries to the lead contact, Duncan S. Palmer: duncan.stuart.palmer@gmail.com.

#### Materials availability

This study did not generate new unique reagents.

#### Data and code availability

All original code has been deposited at Zenodo and is publicly available as of the date of publication (https://doi.org/10.5281/zenodo.10926001). Summary statistics have been deposited at Zenodo (https://doi.org/10.5281/zenodo.10939698) and are publicly available as of the date of publication. DOIs are listed in the key resources table. Quality control and CH calling were performed using Hail (version between 0.2.95 and 0.2.120). Phasing was performed using SHAPEIT5 (5.0.0). All analysis pertaining to PRS were performed using the R-package bigsnpr (version between 1.10 and 1.12.1). Assocation analysis was performed usign SAIGE (1.1.9). Age-of-onset analysis and Kaplan-Meier plotting was performed using R-packages survival (3.2.13) and survminer (0.4.9). Essential and non-essential gene lists were downloaded from https://github.com/macarthur-lab/gene_lists (https://doi.org/10.5281/ZENODO.6724345). Unless otherwise indicated, analyses were performed in R (4.1.1) and python (3.6.13) and plotted using the R-package ggplot2 (3.4.0).

### Method details

#### Exome sequencing quality control summary

We perform a series of hard-filters on genotype, sample, and variant metrics (Tables S22–S24). We confirm genetic sex with reported sex, and restrict analysis to genetically ascertained samples of NFE ancestry, using random forest (RF) classifiers (Figures S25 and S26). Finally, we filter based on a second collection of sample and variant filters (Tables S23 and S24). We used Hail 0.2^45^ and PLINK 1.9^53^ to perform all QC steps, and use R (4.0.2) scripts for plotting and filtering. Data was manipulated in R using data.table (1.14.2) and dplyr (1.0.7), random forest classifiers were trained using the randomForest (4.6–14) library, and plotting was performed using a ggplot2 (3.3.5).

#### Exome sequencing quality control

##### Sample filters

We evaluated sample-level quality control (QC) metrics on the 200,643 UKBB ES multi-sample project level variant call format (VCF) call-set files,^45^ Table S22. All metrics were calculated for bi-allelic single nucleotide polymorphisms (SNPs), except for metrics involving insertions and deletions. We regressed out the first 21 principal components (PCs),^54^ and filtered out sample outliers of the residuals for each metric based on MAD (median absolute deviation) thresholds (Table S22). Samples without PC data were subject to more stringent thresholds (Table S22).

##### Variant-level filters

Retain variants satisfying all of the following conditions.

(1) Not in a low complexity region (LCR).^55^
(2) In sequencing target regions ±50 base pairs.
(3) MAF > 0 following genotype QC.
(4) Excess heterozygosity (ExcessHet <54.69) filter: Phred-scaled P-value for exact test of excess heterozygosity^56^ in founders as determined by relatedness estimates and recorded ages of UKBB participants.^54^ Variants were retained as recommended in genome analysis toolkit (GATK)^56^

##### Genotype filters

Multi-allelic variants were split into bi-allelic variants and insertions and deletions (indel) were left-aligned.^46^ Genotype calls meeting any of the following criteria were set to missing.

(1) Genotype quality (GQ) ≤ 20.
(2) Total sequencing depth (DP) ≤ 10.
(3) Heterozygous calls:
  (a) SNPs: 1-sided binomial test of alternate allele depth related to total read depth -3P<1×10
  (b) Indels: alternate allele read depth/total read depth <0.3.
(4) Homozygous indel calls: alternate allele read depth/total read depth <0.7.

#### Additional ES quality control

To perform further QC we use Hail, an open-source Python library which focuses on the analysis of large-scale genetic datasets. We used Hail to create our own methods, and we take advantage of the functionality that has been rewritten to enable fast and scalable analysis of large exome and genome sequencing projects. Unless otherwise stated, all of the following the data curation and quality control steps were performed in Hail.^45^

Briefly, we apply a collection of hard-filters on sample metrics. We confirm genotypic sex with reported sex, remove samples with excess glsplurv, and restrict analysis to samples of genetically ascertained NFE ancestry. Finally we apply a second collection of sample and variant hard filters. As an initial pass to remove low quality and contaminated samples, we filter out samples with call rate <0.95, mean DP <19.5× or mean GQ <47.8 (Figure S27).

#### Sex imputation

To confirm participant sex and calculate PCs, we extracted high quality common variants (allele frequency between 0.01 and 0.99 with high call rate (> 0.98)) and LD prune to pseudo-independent SNPs using –indep 50 5 2 in PLINK 1.9. When reported sex does not match genotypic sex, it may signal potential sample swaps in the data. Using the F-statistic for each sample using the subset of the non-pseudo autosomal region on chromosome X, we identify and remove samples where reported sex information is not confirmed in the sequence data (Figure S25). Specifically, we remove samples satisfying at least one of the following criteria:

• Sex is unknown in the phenotype files.
• F-statistic > 0.6 and the sex is female in the phenotype file.
• F-statistic < 0.6 and the sex is male in the phenotype file.
• F-statistic > 0.6 and number of calls on the Y chromosome is < 100.

#### Defining samples with non-Finnish European ancestry

To ensure adequate case-control for as many traits as possible, we restricted our analysis to a set of genetically ascertained NFE samples. To do this, we perform a number of principal component analysis (PCA) steps to ensure that we have subset down to NFE. We first run PCA on the 1000 Genomes (1KGP) samples (minus the small subset of related individuals within the 1KGP) using subsetting to LD pruned autosomal variants. We then project in the UKBB samples, ensuring that we correctly account for shrinkage bias in the projection.^57^ Next, we removed samples outside of the European population (EUR) using an RF classifier: we train an RF on the super-populations labels of 1KGP and predict the super-population for each of the UKBB samples (Figure S26). We denote strictly defined European subset as those with probability >0.99 of being European according to the classifier. Another RF classifier is trained following restriction of the 1KGP samples to Europeans to determine NFE, using a classifier probability of 0.95. RF classifiers were trained using the randomForest (4.6) library in R. Samples not assigned to the NFE cluster were removed from downstream analysis.

#### Final hard filters

For our final variant filtering step, following restriction to the NFE subset, and removal of incorrectly defined sex or unknown sex, and run variant QC. We then filter out variants with call rate < 0.97, variants out of Hardy-Weinberg equilibrium (HWE) ($P<1\times 10^{-6}$), and remove invariant sites following the previous sample based filters. After restricting to these high quality variants, we perform a final set of sample filters to finalize the quality controlled data. We evaluate a collection of sample metrics and remove samples falling outside four standard deviations (SDs) of the sequencing batch mean (Ti/Tv, Het/HomVar, Insertion/Deletion ratios), and remove the collection of samples with over 175 singletons. The resultant curated analysis ready dataset consists of 176,935 samples, and 9,169,408 variants (Tables S23 and S24). A summary of sample and variant filters are provided in Tables S23 and S24 and Figure S28. The high quality ES call-set consisted of 176,935 samples and 9,169,408 variants.

#### Phasing

##### Combining ES data with genotype array data

We combined genotyping array (UK BiLEVE Axiom array and UKBB Axiom array) and exome chip (IDT xGen Exome Research Panel v1.0) variants after general ES quality control using Hail^45^ and BCFtools^58^ (1.12). For variants in both datasets, we preferentially retained those on the ES data. For variants on the genotyping array we excluded variants missingness > 5% after performing a liftover to GRCh38 using Hail.^45^ To avoid biasing the phasing quality estimates, we excluded parents among trio relationships prior to phasing. We first created a common variant scaffold by phasing variants in the combined (exome sequencing and genotyping array) data with MAF >0.1% and otherwise default parameters using SHAPEIT5_PHASE_COMMON module. We then phased the remaining rare variants using the common variant scaffold using the SHAPEIT5_PHASE_RARE with recommended parameters. To ensure computational tractability, we phased overlapping chunks of 100,000 variants with ≥50,000 variant overlap between consecutive chunks using Hail.^45^ Following chunk phasing, we then removed the initial and final 22,500 variants from each chunk, so that 5,000 overlapping variants remained between contiguous phased chunks. We then combined the phased chunks, matching haplotype phase using bcftools^58^ (1.12) with the –ligate option. We then restrict this phased genetic dataset to the set of samples and variants present in the analysis ready NFE subset (Tables S23 and S24).

#### Trio-switch error rates

We assessed phasing quality by comparing statistically phased genotypes to those implied in 96 trios using Mendelian inheritance logic. Switch errors are determined by traversing the statistically phased and parent-offspring transmitted haplotypes simultaneously and scanning for inconsistencies in phase between pairs of contiguous variants. This method only allows us to consider sites in which the one parent is heterozygous and the other is homozygous for the reference or alternate allele, and thus do not consider *de novo* variants or Mendelian inconsistencies in the trio data. To assess switch error in a site-specific manner, we modified and recompiled bcftools^58^ (1.12) to output errors by genomic position. We then used the modified version to assess switch by variant categories, for example by genetic data modality (genotyping array or ES), or by MAF bins. To evaluate switch errors across different phasing confidence thresholds, we filtered VCF using Hail^45^ and then repeated the switch error calculation step. We calculated binomial 95% confidence intervals (CIs) for SERs using the R-package HMisc^59^ (4.7).

#### Read-backed phasing

We performed read-backed phasing with UKBB ES short paired-end read sequences using.cram files provided by UKBB. As WhatsHap^27^ (1.6) is computationally expensive, we restricted our analysis to pairs of variants on chromosomes 20–22 in 176,586 genetically ascertained NFEs. We phased both single nucleotide polymorphism (SNV) and indel with WhatsHap using the default recommended parameters. WhatsHap outputs lists of phased variants within ‘phased sets’. We carried forward reads overlapping no more than two variants, for which phase could be inferred. We combined these phased variants with statistically phased variants from SHAPEIT5 using Hail,^45^ and determined agreement between estimated phasing in WhatsHap and SHAPEIT5 (Figure 3).

#### Phenotype curation

We considered a collection of 282 binary quality controlled and publicly available common complex phenotypes for analysis.^60^ To complement these, we also considered 28 common complex phenotypes that were obtained through manual curation, resulting in a total of 311 binary phenotypes for analysis. To increase our power for analyses for binary traits, we amalgamated a collection of phenotypes where possible: combining the phenotype curation of Censin et al.,^61^ with the primary care mappings file provided by UK Biobank all_lkps_map_v3.xlsx and our own manual curation. We aggregated across ICD-10, ICD-9, operating codes, nurses interview reports, and self-reported diagnosis by doctor from the main phenotype file, as well as v2 and v3 read codes in the primary care data. As in Censin et al., we made use of the careful definitions of Eastwood et al.*,*^47^ subsequently applied by Udler et al.^62^ for diabetes subtype curation. Briefly, the algorithm developed in Eastwood et al. bins individuals into putative diabetes status using a collection of phenotypes in the UK Biobank data including self-reported diabetes diagnosis, age of diagnosis, medications, start of insulin within a year of diagnosis. We defined cases as those placed in the probable and possible case categories in the algorithms output. Controls were defined as samples labeled as ‘diabetes unlikely’ by the algorithm.

#### Variant annotation masks

We annotated coding variation using Variant Effect Predictor (VEP)^63^ (v95) using the worst consequence by gene within ‘canonical’ transcripts. We classified variants into four categories: protein truncating variants (PTVs), missense variants, synonymous variants, and other variants (Table S6). We then split PTVs into putative loss of function (pLoF) (HC) and LC loss-of-function variants using LOFTEE,^64^ and labeled missense variants with both Rare Exome Variant Ensemble Learner (REVEL)^65^ score ≥0.6 and CADD^66^ score ≥20 as ‘damaging missense’ or otherwise as ‘other missense’. Finally, we combine the resultant ‘damaging missense’ category with LC loss-of-function variants, which we denote as ‘damaging missense/protein-altering’.

#### Bi-allelic encoding and recessive models

Using custom Hail scripts, we define and annotate individuals as being ‘bi-allelic’ for a gene if they harbor at least one pLoFs or damaging missense variant with MAF < 5% on both inherited copies of the gene. For each sample, we encoded the presence and absence of a damaging bi-allelic variant for each gene as zero and two, respectively. We encode this information in a.vcf file and test for an association between presence of a damaging bi-allelic variant in a gene and a trait using SAIGE (1.1.9),^67^ adjusting for sex, age, sex×age, age ^2^, UKBB center, genotyping batch and the first 10 PCs. We took relatedness into account using a sparse genetic relatedness matrix (GRM) fitted on NFE. We restrict analysis to (gene, trait) pairs with at least five bi-allelic variants in the curated ES with non-missing corresponding phenotype data (corresponding to a minimum MAC ≥10), and adjust for multiple testing at Bonferroni significance (P<0.05/gene−traitpairs).

#### Gene copy dosage encoding and additive models

We define annotate individuals as being ‘mono-allelic’ for a gene if they harbor at least one pLoFs or damaging missense variant with MAF <5% on a single copy of the gene. Furthermore, if they harbor at least one pLoF or damaging missense variant on both inherited copies of the gene, we annotate them as ‘bi-allelic’. Using custom Hail scripts, we encode wildtypes, mono-allelic and bi-allelic carriers as 0, 1 and 2 respectively, thus representing the number of affected gene copies in an individual. We test for association using SAIGE,^67^ adjusting for sex, age, sex×age, age ^2^, UKBB center, genotyping batch and the first 10 PCs. Again, we took relatedness into account using a sparse GRM fitted on NFE. We restricted to gene-pairs with at least 10 disrupted haplotypes (corresponding to a minimum MAC ≥10), and adjust for multiple testing at Bonferroni significance (P<0.05/gene−traitpairs).

#### Polygenic risk scores

##### Curation of array-based genetic data

We generated PRSs using imputed genotypes provided by UKBB.^54^ In the following, we make the distinction between training and testing data. The first represents the samples that are used for fitting LDPred2^68^ weights and parameters while the latter represent the samples with bi-allelic variant (with homozygous or CH status) information in which we use to assess the predictive accuracy the fitted LDPred based PRS. For the training data, we took the genetically ascertained NFE and filtered to 246,152 unrelated samples (kinship coefficient $<2^{-4.5}$) that did not have quality controlled ES data available. NFE samples with high quality ES and imputed genotype data available were used for testing. Where predictive (nominal significant $h_{\text{snp}}^{2}$ and $n_{eff}\geq 5000$), we include PRS as a covariate for downstream biallelic association testing to account for common variant polygenic risk for the trait under investigation.

##### Genotype variant filtering

We followed best practices from Privé et al.*,*^68^ and filtered to common Haplotype Map 3 (HM3) SNPs.^69^ Additionally, we exclude any variants with genotyping proportion <1% and MAF <1%, resulting in a total of 1,165,296 common autosomal variants for fitting PRS weights. To reduce the likelihood of spurious correlations between low-frequency variants in traits with low case or control count, we restricted to binary phenotypes with at least 1,250 cases and controls. Additionally, we imposed a phenotype specific MAF filter based on the number of cases and controls in a trait, specifically:

(Equation 1) $$\text{MAF}>\max\left(0.01,2\times \min\left(n_{\text{cases}},n_{\text{controls}}\right)\right)\text{,}$$

where $n_{\text{cases}}$ and $n_{\text{controls}}$ are the numbers of cases and controls with high quality imputed sequence data available, respectively, to guard against non-causal variants that are overrepresented in cases or controls leading to false positive associations.

#### Common variant association testing

We tested for associations between the 1,165,296 common autosomal HM3 variants and phenotypes using Hail,^45^ running logistic regression (logistic_regression_rows) adjusting for sex, age, sex×age, age ^2^, UKBB assessment center, genotyping batch and the first 10 PCs, using a Wald test.

#### Estimating heritability

We generated LD-scores for HM3 variants in sample, using a random subset 10,000 of 246,152 unrelated genetically ascertained NFEs without haplotype information. Using the genome-wide association study (GWAS) summary statistics and LD-scores, we estimated SNP heritability $h_{\text{snp}}^{2}$ and standard errors (SEs) using LD score regression (LDSC).^48^^,^^70^ We evaluated PRS for phenotypes with nominal significant $h_{\text{snp}}^{2}$ estimates (P<0.05) and restricted to phenotypes with nominally significant (P<0.05) LDSC based SNP heritability estimates and effective sample size $n_{\text{eff}}\geq 5,000$, where:

(Equation 2) $$n_{\text{eff}}=\frac{4}{\frac{1}{n_{\text{cases}}}+\frac{1}{n_{\text{controls}}}}\text{.}$$

#### Generating PRS using LDPred2

For a given phenotype, we trained a PRS predictor with LDPred2-auto,^68^ using marginal effect size estimates evaluated on the 246,152 unrelated NFE samples (defined by kinship coefficient $<2^{-4.5}$) without ES data in the 200k ES UKBB release), $h_{\text{snp}}^{2}$ as estimated by LDSC, and in-sample reference panel to evaluate local LD, as input. We removed any invariant sites and mean-imputed missing genotypes, before training the predictor. Following PRS training, we then predict into the 176,266 samples with ES and high-quality imputed genotype data.

#### Validation of polygenic risk scores

We assessed the ability of the resulting PRS to discriminate between case status by evaluating area under the curve (AUC) on the held-out unrelated set of samples with both HM3 SNPs and phased exome data. We used the function AUCBoot from the R package bigstatsr^49^ (1.5.6) to extract 10,000 bootstrap replicates of individuals and compute the 95% CIs for AUC.

#### Conditional analysis

##### Off-chromosome PRS conditional analysis

For each chromosome, C, we evaluated ‘off-chromosome’ PRS by setting weights on chromosome C to 0. We repeated this for each phenotype with PRS available and fit SAIGE^67^ models while controlling for off-chromosome PRS by including it is as a covariate in the null SAIGE model.

##### Common variant conditional analysis

To assess whether a putative signal in a gene is driven by nearby common variation, we filtered to samples that have both ES and imputed genotypes with MAF >1% and imputation INFO score >0.5. Then, for each gene that passed exome wide significance in the primary analysis ($P<5\times 10^{-6}$), we tested for common variant associations in the region (1 Mb upstream and downstream of the gene). For each of these regions, we took an iterative approach, testing for common variant associations using SAIGE,^67^ conditioning on the lead variant and repeating the regression until the conditional P for the newly included variant dropped below $5\times 10^{-6}$, allowing up to 25 ‘independent’ associations in the region. We used the same covariates as in the primary analysis. For every variant that passed exome-wide significance ($P<5\times 10^{-6}$), we encoded the genotypes as dosages and embedded them alongside pseudo variants (bi-allelic variants) in a VCF. We then re-ran the primary analysis twice (with and without controlling for off-chromosome PRS), while conditioning on any nearby common variant signals of association with the phenotype of interest.

##### Rare and ultra-rare variant conditional analysis

For each significant ($P<1.68\times 10^{-7}$) gene-trait associations in the genome-wide analysis after conditioning on PRS and nearby common variants association signals, we considered a further conditioning step. We sought to determine whether the residual signal of association could be explained by additive rare variant effects within the associated gene. To do this, ran further conditioned on rare (MAC ≥10, MAF ≤0.05) and ultra-rare (MAC ≤10) variants annotated as either pLoF or damaging missense within each gene. Because conditioning on ultra-rare variation can lead to convergence issues, we performed a gene-wide collapsing of ultra-rare (MAF ≤10) variants, thus aggregating them into a single ‘super’ variant to represent burden of ultra-rare damaging variation in the gene. Following this collapsing, we were able to condition on the ultra rare and rare variant contribution using SAIGE, while also conditioning on PRS and nearby common variant association signals when applicable.

#### Permutation of genetic phase

To test whether a putative gene-trait association is driven by compound heterozygosity, we designed a permutation-based pipeline that could be systematically applied and scaled across phenotypes and genes. To do this, we label samples that are either CH variants or heterozygous *cis* carriers and then randomly shuffle these labels a series of times. For each permutation, we re-run the association analysis conditioning on covariates as previously discussed (including off chromosome PRS and nearby common variants), and determine the resultant association strength under this label shuffling. Applying this permutation procedure multiple times, we can determine an empirical null for the association strength in the absence of phase information. The result is an empirical distribution of score-statistics and corresponding P-values that reflect the degree of association that would be expected given that the phase is random. We evaluate the one-sided empirical P-value, specifically:

(Equation 3) $$P_{\text{empirical}}=\frac{1}{n}\sum_{i=1}^{n}I\left(t_{i}\geq t_{\text{observed}}\right)$$

where n is the number of permutations, $t_{i}$ is the score-statistic under the $i^{\text{th}}$ random label shuffling, and $t_{\text{observed}}$ is the observed score-statistic determined using the observed genetic phase. To ensure sampling of score-statistics at a sufficiently large number of configurations of the genetic phase, we analyzed gene-trait pairs with at least ten compound heterozygotes and/or samples with multiple variants on the same haplotype. We permuted up to 100,000 times. To control for multiple testing, we corrected for 5 gene-traits tested (Bonferroni significance threshold P<0.05/5=0.01).

#### Gene-set enrichment of bi-allelic variation

##### Analyzed gene-sets

We included the following gene lists in our gene-set enrichment analyses: essential in mice,^50^ essential gnomAD,^28^ essential ADaM,^51^ essential in culture,^52^ essential CRISPR,^71^ genes with pLI >0.9 in gnomAD,^28^ non-essential in culture,^52^ homozygous LoF tolerant,^28^ and non-essential gnomAD^28^ and Curated Cancer Cell Atlast.^72^

##### Poisson regression to assess enrichment of CH variants in gene-sets

We test for depletion and gene-set enrichment using poisson regression. We model the count of bi-allelic variants across samples as a function of gene-set and mutation frequency using the glm function in R.

(Equation 4) $$\begin{array}{c} \left|\text{samples}\;\text{with}\geq 1\;\text{variant}\;\text{of}\;\text{class}\;x\;\text{in}\;\text{gene}\right|∼I\left(\text{gene}\text{−}\text{set}\right)+\text{mutation}\;\text{rate} \end{array}$$

where x is a pair $\left(x_{1},x_{2}\right)$: $x_{1}\in$ {pLoF, damaging missense, pLoF and/or damaging missense, other missense, synonymous}, $x_{2}\in$ {heterozygote, CH, bi-allelic variants}. For each annotation category we use the transcript-specific mutation rate.^29^ 95% confidence intervals are determined using confint.glm from the MASS-package (v7.3–58.1).

#### Homozygote and CH down-sampling

To investigate the number of identifiable CH or homozygous events across varying sample sizes and variant annotations, we performed down-sampling across the total population of 176,587 individuals. To do this, we defined a set of 35 regularly spaced cutoffs between 1,000 and 176,587 samples using increments of 5000. To determine uncertainty in our estimates of the number of unique genes implicated as a homozygote and/or CH, we randomly sampled individuals for each down-sampling 100 times, with replacement. We calculated the 95% CI by taking the 2.5% and 97.5% quantiles for the number of unique genes affected at a given sample size, and repeated across annotations (Figure S13).

#### Power analysis for bi-allelic association

We perform a power analysis based on bi-allelic (including both CH and homozygous) variant frequencies in the population. To do this, we adopted code^73^ allowing us to determine the effective effect size on the OR scale across candidate configurations of binary case-control counts by substituting alternate allele frequencies with bi-allelic variant frequencies. We calculated effect sizes at 80% power at Bonferroni significance ($P<1.68\times 10^{-7}$) for a hypothetical traits with 823 (0.5%), 1766 (1%), 3532 (2%), 5298 (3%), 8829 (5%) cases of 176,587 total samples.

#### Simulation

##### Simulation of phenotypes using real genotypes

We performed a series of simulations to test that our pipeline would detect a CH effect in the presence of a true signal. We sampled 100,000 genetically-ascertained NFEs in the UKBB data, and extract chromosome 22 which we then use to simulate phenotypic data with a recessive genetic architecture. To emulate a scenario in which defects in protein coding genes lead to disease, we annotated the filtered UKBB genetic data and determined the collection of samples harboring damaging bi-allelic variants in each gene (compound heterozygous and homozygous, comprised of variants annotated as pLoF or damaging missense). We then define a n samples × m genes matrix $\tilde{B}$ with entries:

(Equation 5) $$\begin{array}{c} {\tilde{B}}_{i,j}=\left\{ \begin{array}{cc} 1, & \text{if}\;a\;\text{damaging}\;\text{bi}-\text{allelic}\;\text{variant}\;\text{is}\;\text{present}\;\text{in}\;\text{sample}\;i\;\text{at}\;\text{gene}\;j\\ 0, & \text{otherwise} \end{array}\right. \end{array}$$

We then simulated liability under the following model:

(Equation 6) $$\begin{array}{c} y_{i}=\sum_{j=1}^{m}B_{i,j}\theta_{j}+\varepsilon_{i} \end{array}$$

where $B_{i,j}$ is the ${\left(i,j\right)}^{\text{th}}$ entry of B after standardizing the columns of $\tilde{B}$, $E\left[\theta_{j}\right]=\frac{b}{m}$, $\text{Var}\left[\theta_{j}\right]=\frac{h^{2}}{m}$, and $\varepsilon_{i}∼N\left(0,1-h^{2}\right)$. Here, we implicitly assume that presence of at least one homozygous or CH variant of any type within a given gene contributes the same risk to disease, whose average across genes is set by the parameter b. The resultant liability $y_{i}$ has mean 0 and variance 1. Note that the standardization of B imposes a frequency dependent relationship between prevalence of bi-allelic damaging variants in a gene and variance explained. We simulated under the spike-and-slab model:

$$\begin{array}{l} \theta_{j}∼\left\{ \begin{array}{cc} N\left(\frac{b}{m\pi_{\theta }},\frac{h^{2}}{m\pi_{\theta }}\right), & \text{if}\;p_{j}<\pi_{\theta }\\ 0, & \text{otherwise} \end{array}\right.\\ p_{j}∼\text{Bernoulli}\left(\pi_{\theta }\right) \end{array}$$

in which $\pi_{\theta }\in \left[0,1\right]$ is the proportion of causal genes with a recessive contribution to the phenotype. Finally, to obtain binary traits we used the liability threshold model assuming a case prevalence of 10%. In the following simulations, we set $\pi_{\theta }=0.25$, and considered $h^{2}$ values of $h^{2}\in \left\{0,0.01,0.02,0.05,0.10\right\}$ and b values of b∈{0,0.5,1,2,10}.

#### Age-at-onset analysis

##### Time-to-event data curation

We curated age-at-diagnosis for 278 binary phenotypes from the UKBB-linked primary care and hospital record data. 251 phenotypes were curated using the mapping tables generated by Kuan et al.*,*^60^ excluding any codes related to “history of …” events for which accurate age-at-diagnosis could not be extracted. The remaining 27 phenotypes were left-truncated at the age of first record (of any code) in either the primary care or hospital data, and right-censored at the age of the last record.

#### Cox proportional-hazards modeling

For each gene-trait combination to test, we performed Cox-proportional hazards modeling to estimate differences in lifetime risk of developing the phenotype between heterozygous carriers of pLoF + damaging missense/protein-altering variants in the gene (reference group) and individuals who are bi-allelic carriers (compound-heterozygous or homozygous), multi-hit *cis*-heterozygous carriers, and wildtypes. All effects were adjusted for sex, the first 10 genetic PCs, birth cohort (in ten-year intervals from 1930 to 1970), and UKBB assessment center. For phenotypes with a significantly heritable PRS, we additionally adjusted for off-chromosome PRS. We visualized survival probabilities using Kaplan-Meier curves.^74^ Finally, for gene-trait combinations where we were powered to detect differences between compound-heterozygous and multi-hit *cis* heterozygous carriers of variants, i.e., where each group contained at least five cases of the phenotype, we repeated the above analysis with multi-hit *cis* heterozygous carriers as the reference group. Cox proportional-hazards regression was performed using the R package survival 3.3.1^75^ and Kaplan-Meier plots drawn with the R package survminer 0.4.9.^76^

#### Replication analyses

In our initial analysis, we examined 176k individuals from the initial release of 200k exomes. The subsequent release of the full 450k exomes during the drafting of this paper presented an opportunity to replicate our bi-allelic associations. To do this, we leveraged the gnomAD joint variant called exomes which we combined with genotyping array data for the 450k participants, allowing phasing of rare variants. Before phasing, we performed the same initial quality-control using various hard filters, as in the discovery cohort. Our replication cohort consist of the 233,837 individuals not included in the discovery analysis. In the cross-sectional analysis, we performed replication for genes with at least five bi-allelic (pLoF + damaging missense/protein-altering) variant carriers were present. Similarly, in the longitudinal analysis, we performed replication for gene-trait combinations with at least five bi-allelic variants (homozygotes or CH) and 100 heterozygotes in the population. In the replication cohort, variant annotation masks were created using VEP (v105).

#### Simulation of CH and homozygous variants

We conducted simulations to compare the expected and empirically observed number of bi-allelic variants. We focused on 1,174 genes with at least one homozygous of CH variant in our dataset. Then we generated genotypes for 176,935 individuals. Each genotype simulation assumed that variants were independent and utilized a Bernoulli distribution, where the probability of success was set to the variant’s minor allele frequency. We ran the simulations 10 times and derived the expected number of homozygous or CH events with. This average provided us with an estimate for the expected number of bi-allelic variants, including both homozygotes and CHs. We then compared these estimates to our actual empirical findings to benchmark the concordance.

## Supplemental information

Document S1. Figures S1–S24 and Tables S22–S24

Table S1. Comparative analysis of SER point estimates for different phasing methods, related to Figure 1

Table S2. Trio-SER across final reconstituted chromosomes before and after filtering by phasing confidence, related to Figure 1

Table S3. Trio-SER by MAC bin for 96 parent-offspring trio relationships before and after filtering by PP ≥0.9, related to Figure 1

Table S4. Number of trio-switch errors binned by genes before and after filtering by PP ≥0.9 using 93 trio-offspring relationships, related to Figure 1

Table S5. Comparative analysis of haplotype inference using statistical and physical phasing, related to Figure 1

Table S6. Results from variant annotation before and after filtering to sites with PP ≥0.9, related to Figure 1

Table S7. Gene set enrichment analysis of mono- and bi-allelic variants, related to Figure 1

Table S8. Counts of bi-allelic and mono-allelic carriers identified in the study, related to Figure 1

Table S9. Overview of associations before and after conditioning on off-chromosome PRS, nearby common variation, and the burden of rare variation, related to Figure 2

Table S10. Replication analysis of the Bonferroni significant associations from the cross-sectional analysis, related to Figure 2

Table S11. Results and benchmarks for PRSs, related to Figures 2 and 4

Table S12. Systematic search and conditional analysis of common variants around significant gene-trait associations, related to Figures 2 and 3

Table S13. Overview of Bonferroni-corrected significant hits $\left(p<9.8\times 10^{-9}\right)$ from additive association analysis, related to Figure 2

Table S14. Comparison of additive haplotype-collapsing association analysis and SAIGE-GENE+ burden testing, related to Figure 2

Table S15. Bonferroni significant associations $\left(p<1.89\times 10^{-7}\right)$ in Cox proportional hazards models, related to Figures 4 and 5

Table S16. Cox proportional hazards ratios when comparing CH, homozygous, heterozygous, variants in *cis*, and wild-type label against heterozygous carrier status, related to Figure 4

Table S17. Replication analysis of the Bonferroni significant associations from the age at onset analysis, related to Figures 4 and 5

Table S18. Cox proportional hazards ratios when comparing CH, homozygous, heterozygous, and wild-type label against carriers of multiple variants in *cis*, related to Figure 4

Table S19. Median age of diagnosis across tested phenotypes, related to Figure 4

Table S20. Overview of variant configuration and corresponding allele frequencies in highlighted associations, related to Figures 2 and 4

Table S21. Overview of co-occurring variants within gene-trait combinations from age at onset analysis, related to Figures 4 and 5

Document S3. Article plus supplemental information
