# Supplementary material for: Exome-wide evidence of compound heterozygous effects across common phenotypes in the UK Biobank
Source: Cell Genom. 2024 Jun 28;4(7):100602. doi: 10.1016/j.xgen.2024.100602 (PMC11293579; doi:10.1016/j.xgen.2024.100602)
Supplement: Document S1. Figures S1–S24 and Tables S22–S24 [file mmc1.pdf]

Cell Genomics, Volume *4*

Supplemental information

Exome-wide evidence of compound heterozygous effects across common phenotypes in the UK Biobank

Frederik H. Lassen, Samvida S. Venkatesh, Nikolas Baya, Barney Hill, Wei Zhou, Alex Bloemendal, Benjamin M. Neale, Benedikt M. Kessler, Nicola Whiffin, Cecilia M. Lindgren, and Duncan S. Palmer

Supplemental information for Exome-wide evidence of compound heterozygous effects across common phenotypes in the UK Biobank

Frederik H. Lassen[1,2,*], Samvida S. Venkatesh[1,2], Nikolas Baya[1,2], Barney Hill[2], Wei Zhou[4,5,6], Alex Bloemendal[4,7,8], Benjamin M. Neale[4,5,6], Benedikt M. Kessler[3], Nicola Whiffin[1,2,4], Cecilia M. Lindgren[1,2,9†] and Duncan S. Palmer[2,†,*]

*Correspondence to: flassen@well.ox.ac.uk, cecilia.lindgren@wrh.ox.ac.uk and duncan.stuart.palmer@gmail.com

This file includes:

- Tables **S22-S24**
- Figure **S1-S24**

Metric	Metric residual (w/ PCs)	Raw (w/o PCs)
call_rate	[24, ∞)	[4, ∞)
n_insertion	[8, 8]	[4, 4]
n_deletion	[8, 8]	[4, 4]
r_insertion_deletion	[8, 8]	[4, 4]
n_het	[12, 12]	[4, 4]
n_hom_var	[12, 12]	[4, 4]
r_het_hom_var	[16, 16]	[4, 4]
n_non_ref	[8, 8]	[4, 4]
n_singleton	(-∞, 16]	(-∞, 4]
n_snp	[8, ∞)	[4, 4]
n_transition	[8, 8]	[4, 4]
n_transversion	[8, 8]	[4, 4]
r_ti_tv	[8, 8]	[4, 4]

Table S22: **Sample filtering: MAD Intervals, related to Star Methods**. The interval $[a, b]$ represents $\text{median}(X) + \text{MAD}(X)[-a, b]$ for the metric, X. Samples with metrics outside these intervals were removed.

**Filter**	**Samples**	**Batch 1**	**Batch 2**	**%**
Initial samples in raw UKBB variant call format (VCF)	200,643	NA	NA	100.0
Initial samples in filtered VCF	199,795	49,759	150,036	99.6
Sample call rate <0.95	7,400	4,780	2,620	3.7
Mean DP <19.5	3,253	511	2,742	1.6
Mean genotype quality (GQ) <47.8	2,123	834	1,289	1.1
Samples with sex swap	85	24	61	0.0
Samples with excess ultra-rare variants (URVs)	76	6	70	0.0
PCA based filters	13,537	3,390	10,147	6.7
Within batch Ti/Tv ratio outside 4 standard deviations (SDs)	13	3	10	0.0
Within batch Het/HomVar ratio outside 4 SDs	251	46	205	0.1
Within batch Insertion/Deletion ratio outside 4 SDs	9	4	5	0.0
n singletons >175	19	2	17	0.0
**Samples after all sample filters**	**176,935**	**41,371**	**135,564**	**88.2**

Table S23: **Summary of sample filters, related to Star Methods**. Moving down through the rows of the table, we move through QC filtering steps.

**Filter**	**Variants**	**%**
Initial variants in raw UKBB VCF	15,922,704	100.0
Variants removed in initial filters	2,883,660	18.1
Invariant sites after sample filters	2,744,044	17.2
Overall variant call rate <0.97	1,122,987	7.1
Variants failing HWE filter	5,237	0.0
**Variants remaining after all filters**	**9,169,408**	**57.6**

Table S24: **Summary of variant filters, related to Star Methods**. Moving down through the rows of the table, we move through QC filtering steps

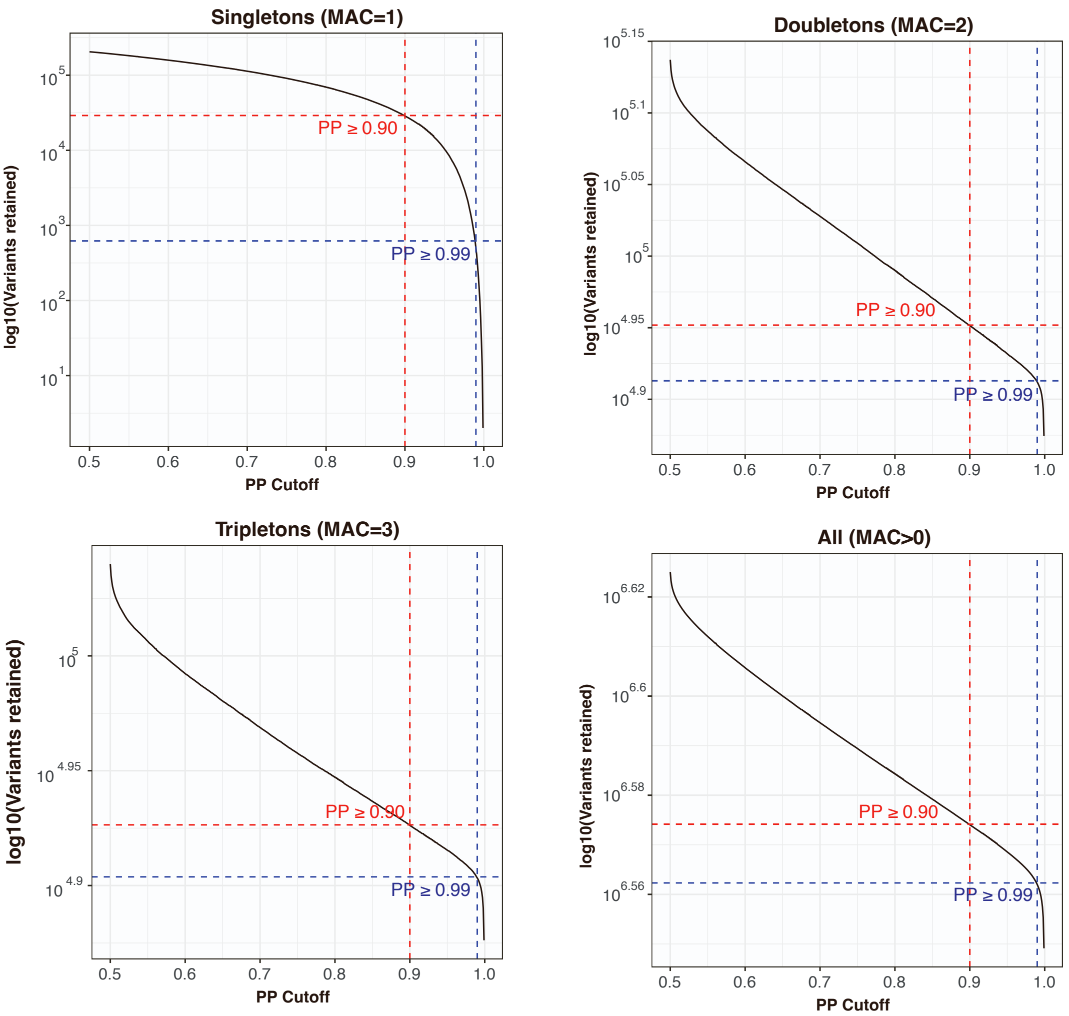

Figure S1: **Phased variants retained as a function of phasing confidence score, related to Figure 1**. Each subplot displays the number of variants retained on the $\log_{10}$ scale as the PP is increased, split by rarity of variants described in the subplot title. Dotted red and blue lines highlight the number of variants retained after imposing PP cut-offs of 0.9 and 0.99, respectively.

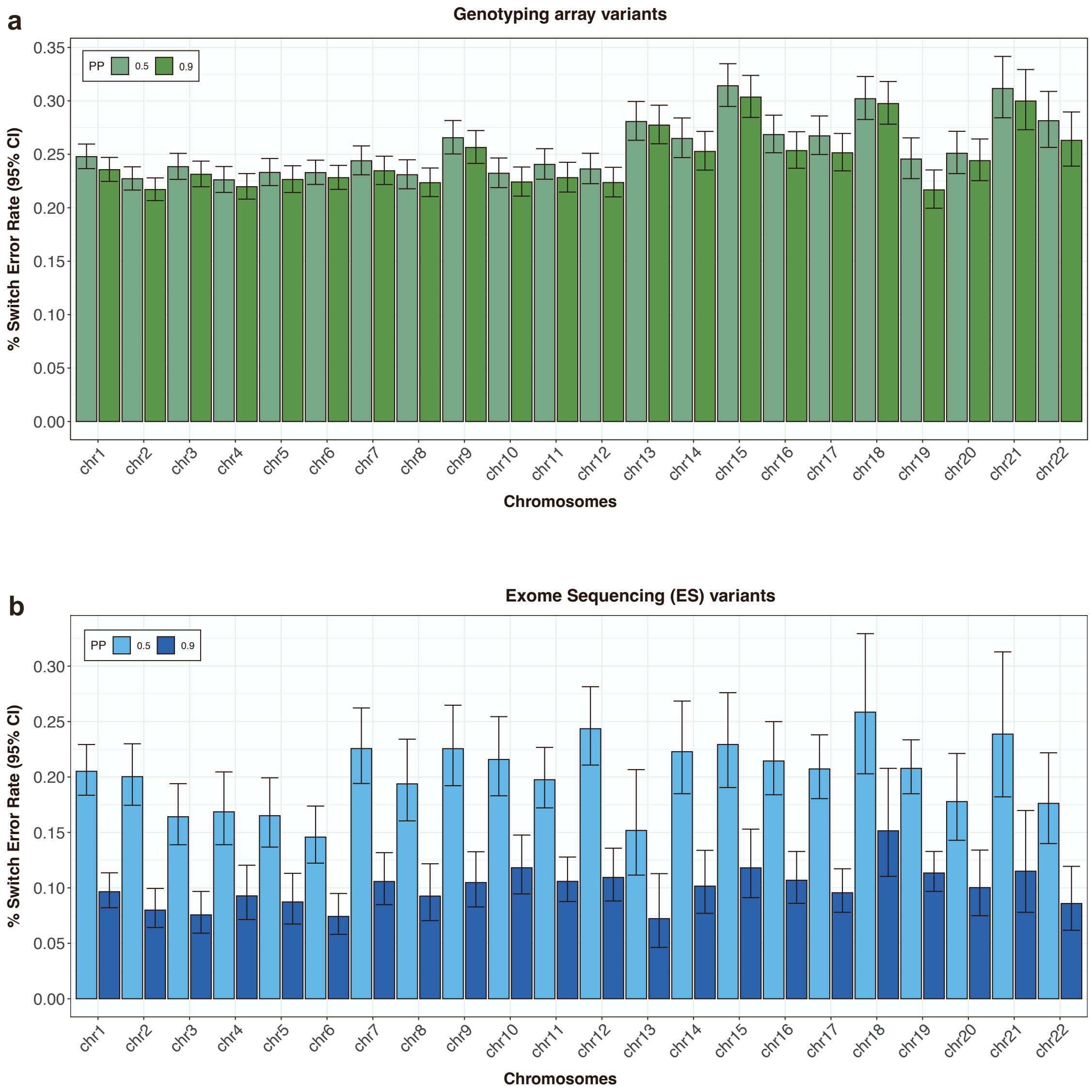

Figure S2: **Trio switch error rates by chromosome, related to Figure 1**. Parent-offspring trios are used to determine switch error rates for variants that originate from the genotyping array **(a)** and exome sequencing data **(b)**. We stratify by phasing confidence (PP) according to the color legends. Mean switch error rates are plotting, with whiskers enclosing the 95% binomial CI.

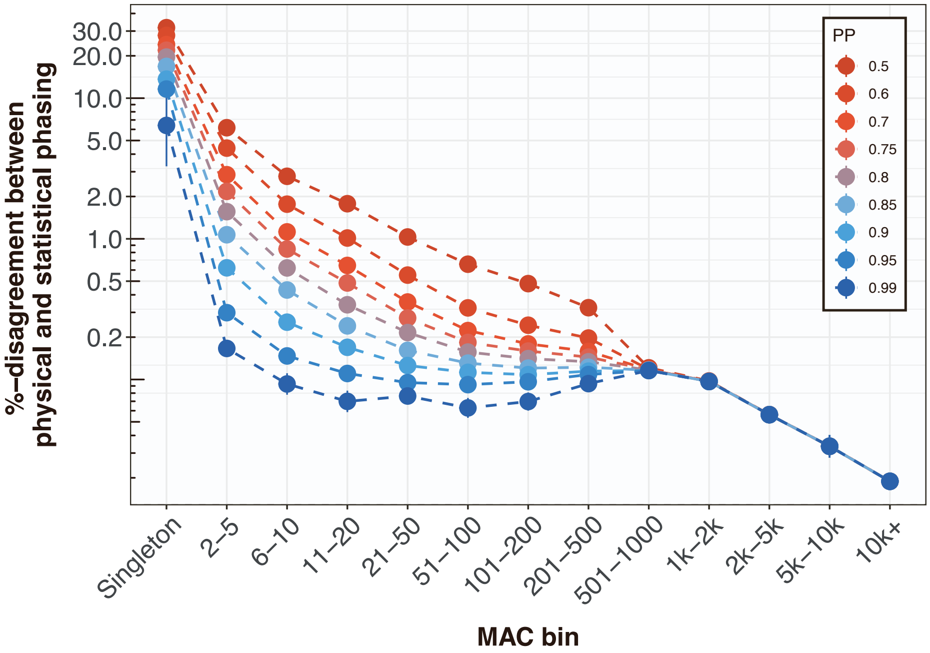

Figure S3: **Agreement between read-backed and statistical phase estimation, related to Star Methods**. Genetic phase was estimated using WhatsHap (read-backed phasing) and SHAPEIT5 (statistical phasing) in 49,756 individuals across all autosomes. We only carried forward pairs of variants close proximity in which phase could be inferred using WhatsHap. We combined with statistically phased counterparts derived from SHAPIET5 and determine % disagreement of phase estimation of variant pairs on the y-axis, when filtering to phased pairs of variants where the minimum PP $> p$ for $p \in \{0.5, 0.6, 0.7, 0.75, 0.8, 0.85, 0.9, 0.95, 0.99\}$ according to the color legend. We stratify pairs of variants into bins based on the minimum MAC in the variant pair, on the x-axis. Mean disagreement rates are plotted on y-axis with whiskers enclosing the 95% binomial CI

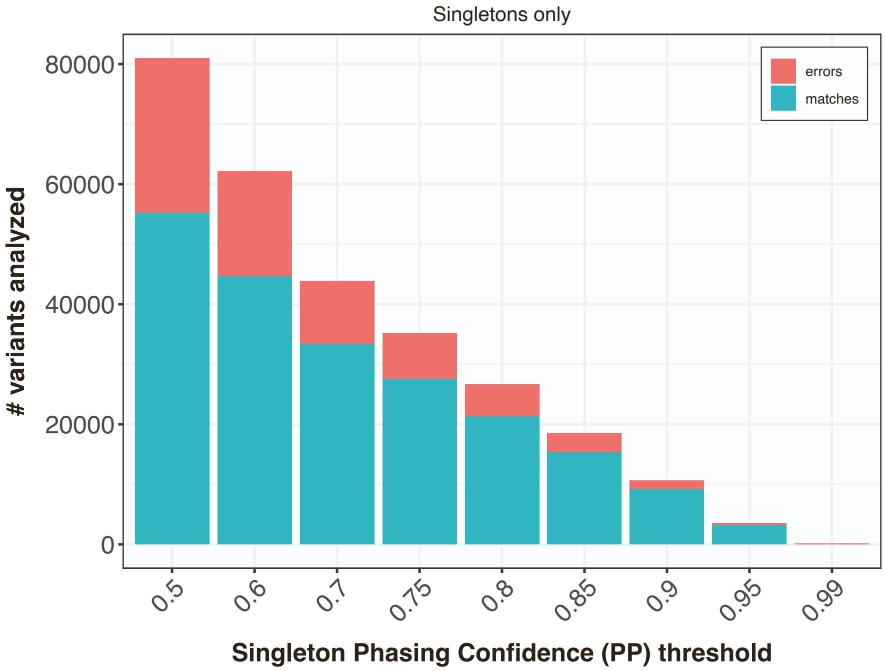

Figure S4: **Agreement between read-backed phased and statistically phased singleton variants, related to Figure 1**. Across the 49,756 samples, we identified 80,978 reads with at least one singleton (MAC= 1) variant irrespective of phasing quality. For each PP-bin we determine the agreement between read-backed phased variants and statistically phased variants with red indicating disagreement and blue indicating agreement. With higher PP-score, the proportion of correctly phased variants increases, while the total number of variants assessed decreases.

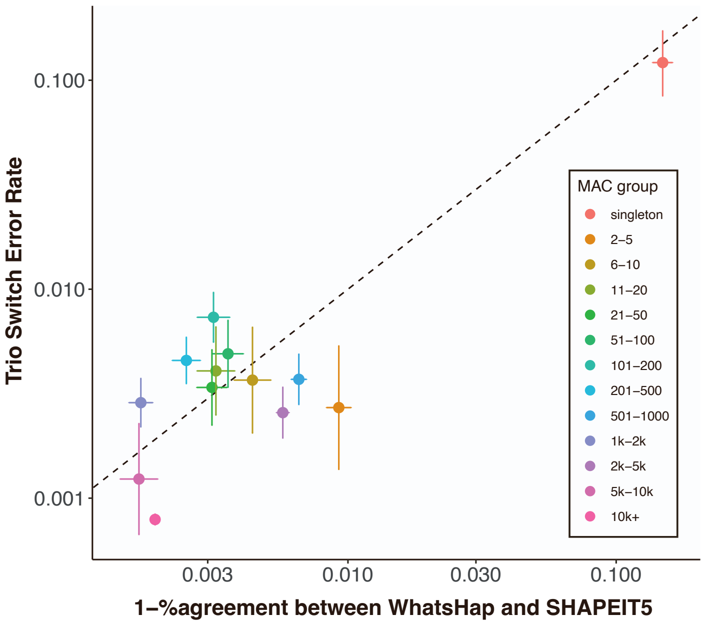

Figure S5: **Agreement between read-backed phasing and statistical phasing, related to Star Methods**. We plot the disagreement between WhatsHap (read-backed phasing) and SHAPEIT5 (statistical phasing) in UKBB on the x-axis against switch error rate in SHAPEIT5 phase estimates implied by trio-based phasing in UKBB on the y-axis. For each comparison, bin pairs of variants according to the minimum MAC in the variant pair according to the color legend. Horizontal and vertical lines enclose 95% binomial CIs around mean estimates. The dotted line is included to display $y = x$.

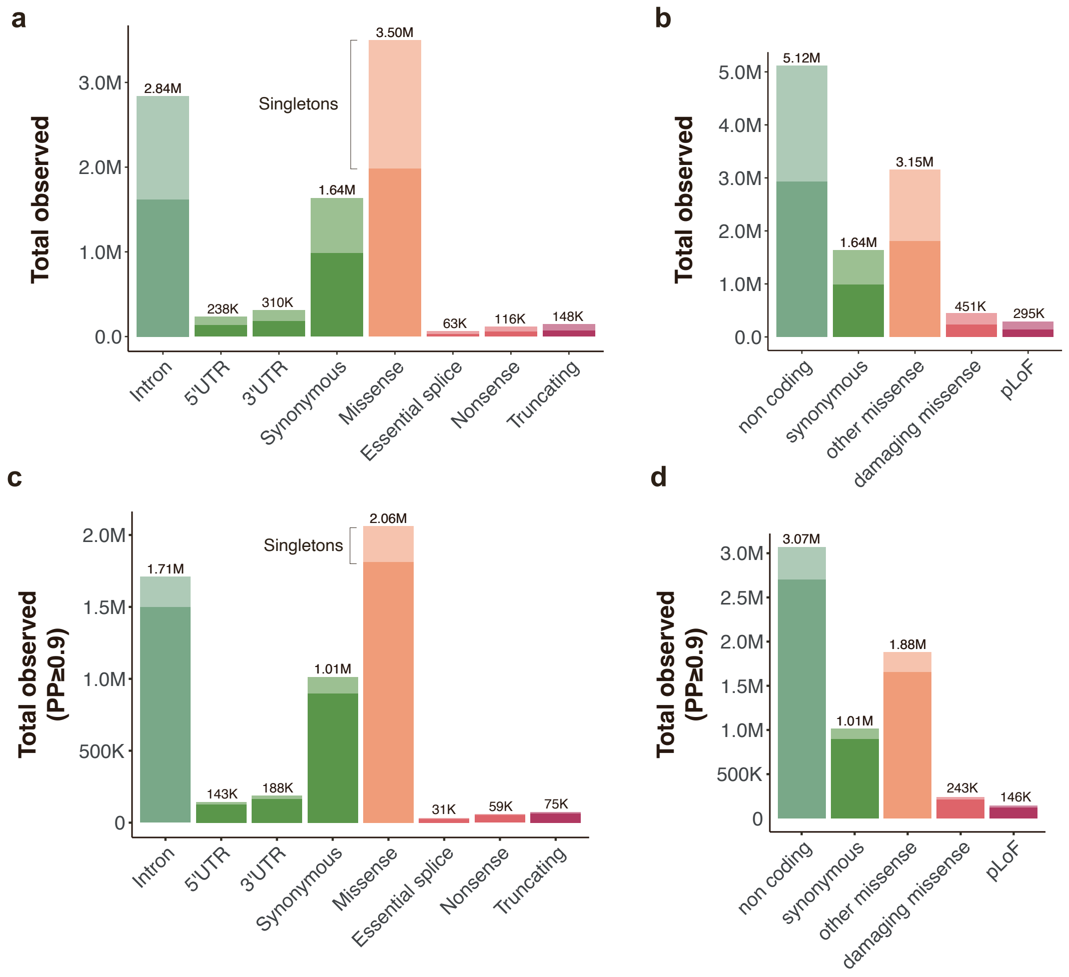

Figure S6: **Distribution of variant annotation categories before and after broad consequence categorization and before and after filtering by PP$\geq$ 0.9, related to Figure 1**. We annotate variants using VEP and by the most severe consequence in the canonical transcript. Panels **(a)** and **(b)** display the total number of unique variants observed across a set of variant consequences colored by degree of predicted impact, before and after broad variant consequence categorization. Panels **(c)** and **(d)** depicts the same as above but after restricting to variants with PP$\geq$ 0.9. In each panel, green, orange and red colored bars indicate low, medium and high impact respectively, according to the color legends. Singleton variation within the variant class is stacked and displayed in a lighter shade. Counts of variant within each annotation category are displayed above the bars. Note that all counts shown here are before filtering to accurately phased variants.

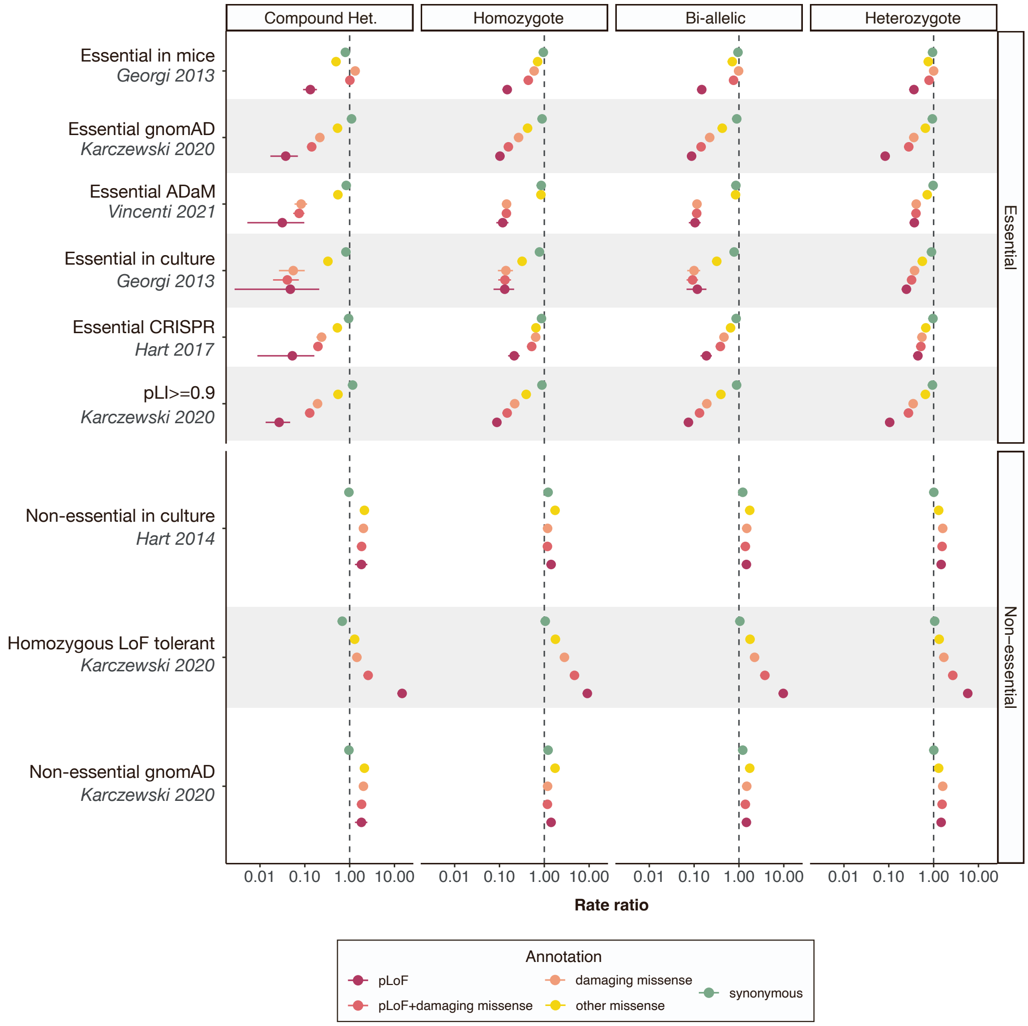

Figure S7: **Gene-set depletion/enrichment modeling, related to Star Methods**. Poisson regression to model mono- and bi-allelic variant (heterozygous, CH, homozygous or both) depletion and enrichment across essential and non-essential gene-sets. Rate ratios are shown for synonymous (green), other missense (yellow), damaging missense (orange) and pLoF (red) variants. The dashed line depicts a rate ratio of 1.

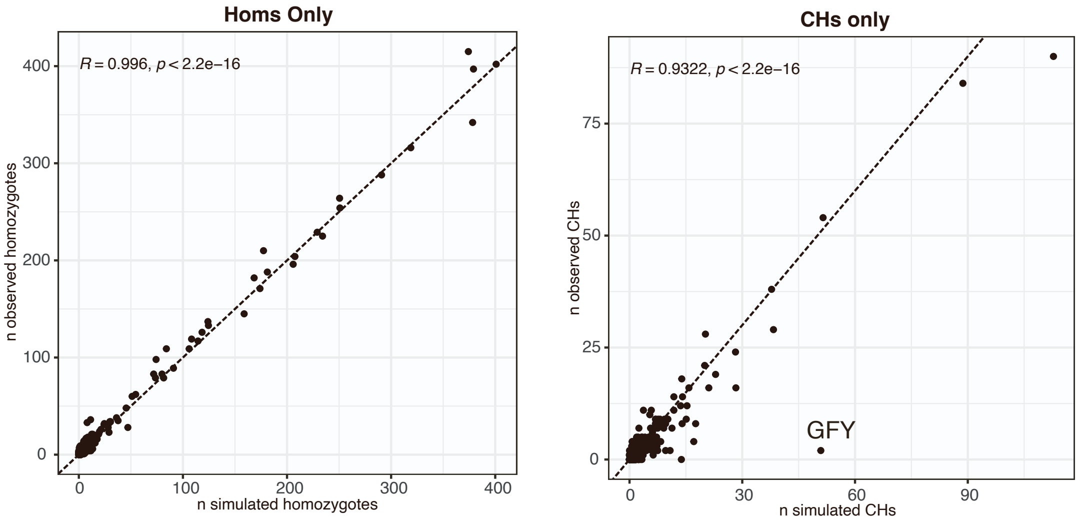

Figure S8: **Simulation of CH and homozygous events in an outbred population, related to Star Methods**. We generated genotypes for 1174 genes using allele frequencies derived from observed pLoF variants. For each gene, we simulated genotypes for 176,935 individuals. We averaged the number of bi-allelic variants across 10 simulations. This served as an estimate for the expected count (x-axis) of bi-allelic variants, against which we compared the empirically observations (y-axis). The first panel (left) is the comparison between observed and simulated homozygous variants. The second panel (right) is the comparison between observed and simulated CH variants.

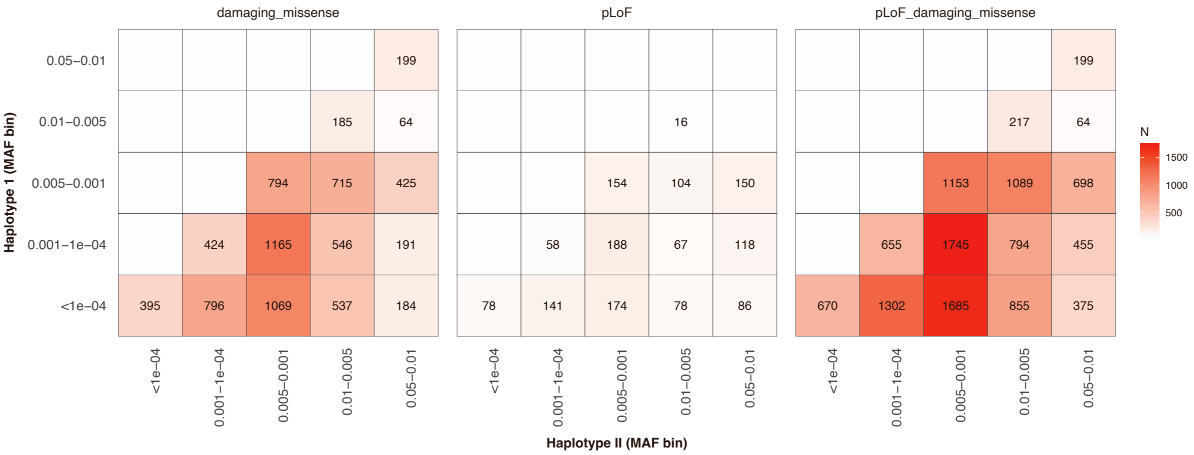

Figure S9: **Allele frequencies of variants in the CH state, related to Star Methods**. Heatmap of allele counts for variants in CH state stratified by predicted variant consequence (damaging missense, pLoF or pLoF+damaging missense). We plot the MAC for variants residing on the most common haplotype (y-axis) versus the rarest haplotype (x-axis).

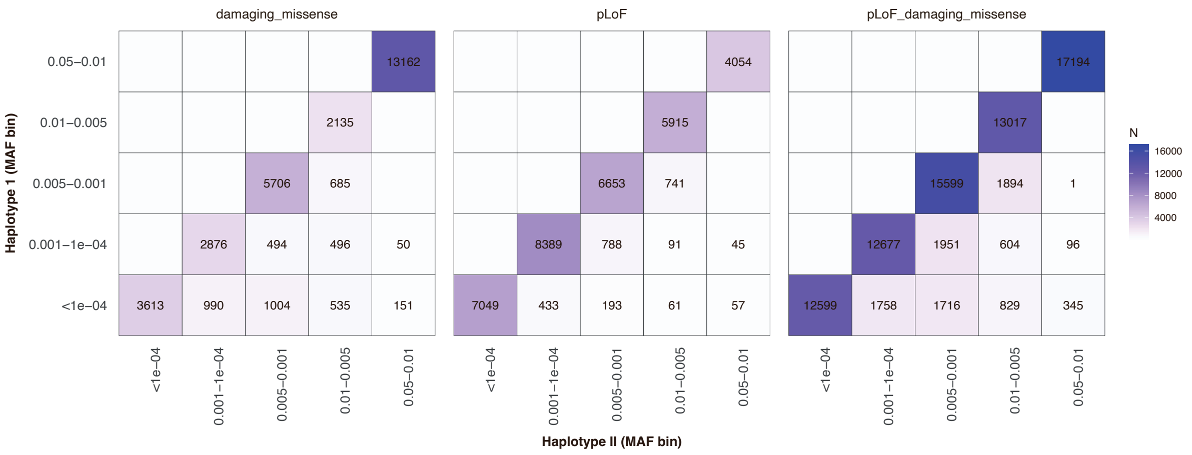

Figure S10: **Allele frequencies of variants in *cis*, related to Star Methods**. Heatmap of allele counts for co-occurring variants on the same haplotype stratified by predicted variant consequence (damaging missense, pLoF or pLoF+damaging missense). The most common variant on the haplotype versus the least common are plotted.

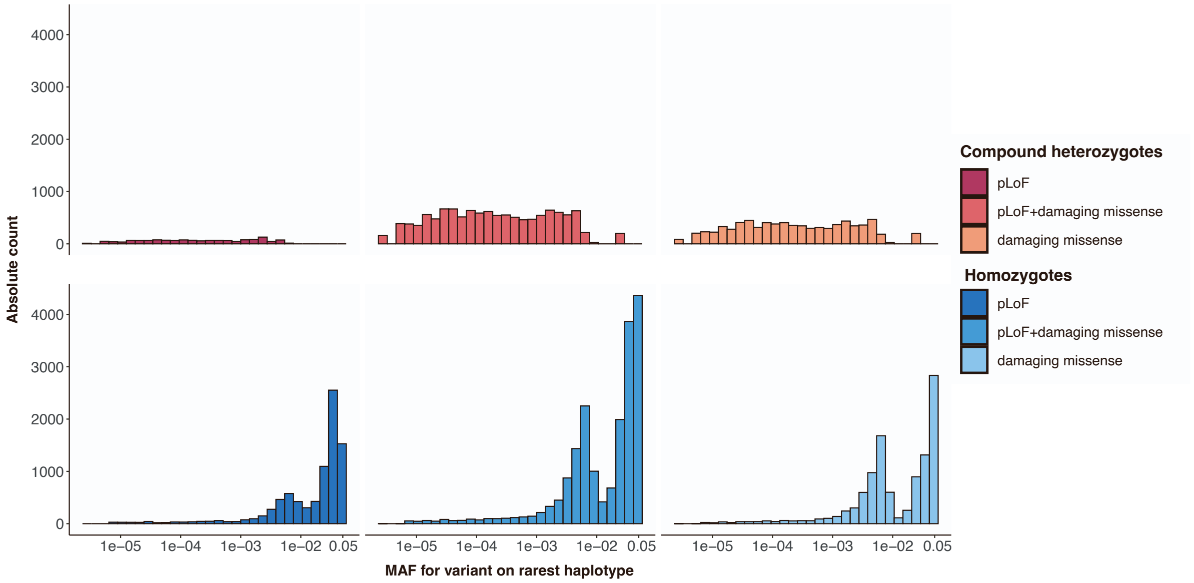

Figure S11: **Distribution of observed variants across samples by allele frequency, related to Star Methods**. Histogram of unique bi-allelic variant (CH and homozygotes) prevalence across the allele frequency spectrum. For a qualifying CH variant, the allele frequency corresponding to the alternate allele on the rarest haplotype are plotted.

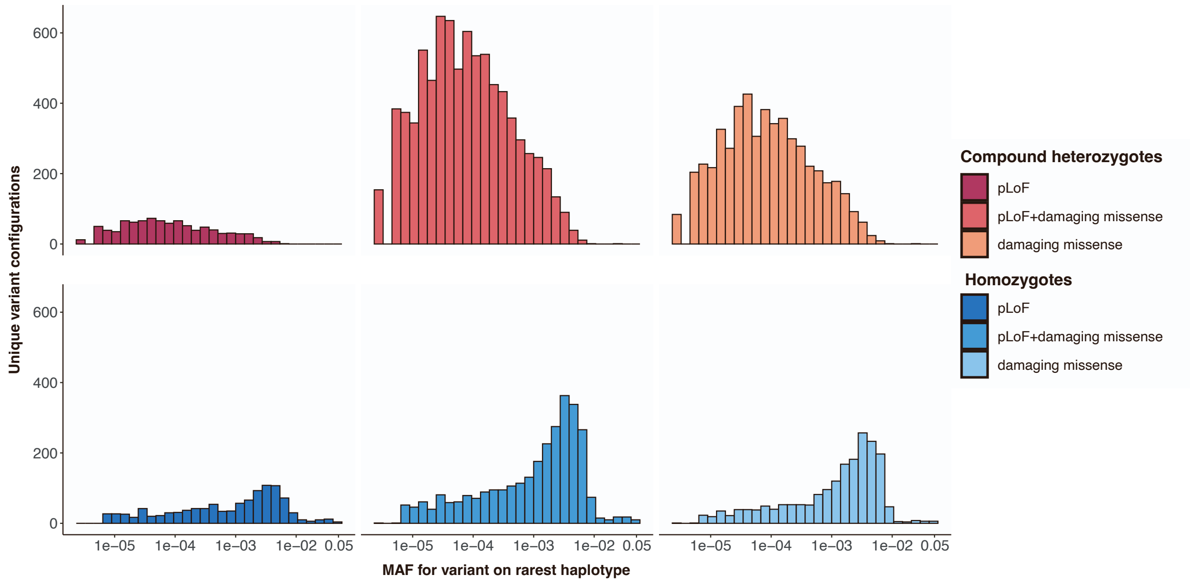

Figure S12: **Distribution of unique variants observed by allele frequency, related to Star Methods**. Histogram of bi-allelic variant (CH and homozygotes) count for all gene-samples pairs in the analysis. For a qualifying CH variant, the allele frequency corresponding to the alternate allele on the rarest haplotype are plotted.

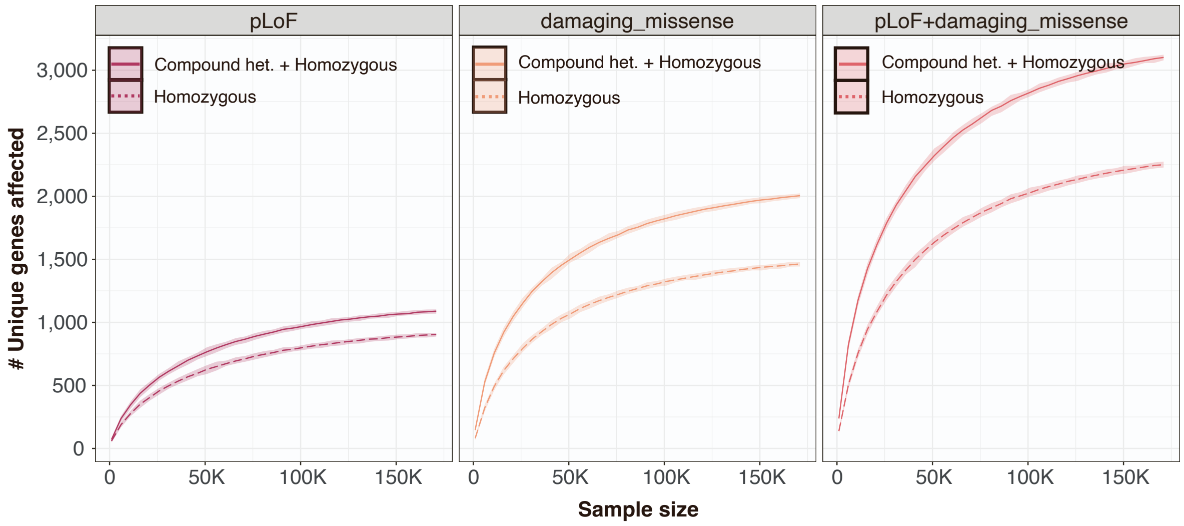

Figure S13: **Count of unique genes affected by a homozygous and homozygous or compound heterozygous variants as a function of sample size, related to Star Methods**. Starting with the full data, and down-sampling, we plot counts number of unique genes harboring homozygous and homozygous or compound heterozygous variants as a sample size is decreased. Class of variants in each count are denoted according to the key. Each facet indicate a specific variant annotation.

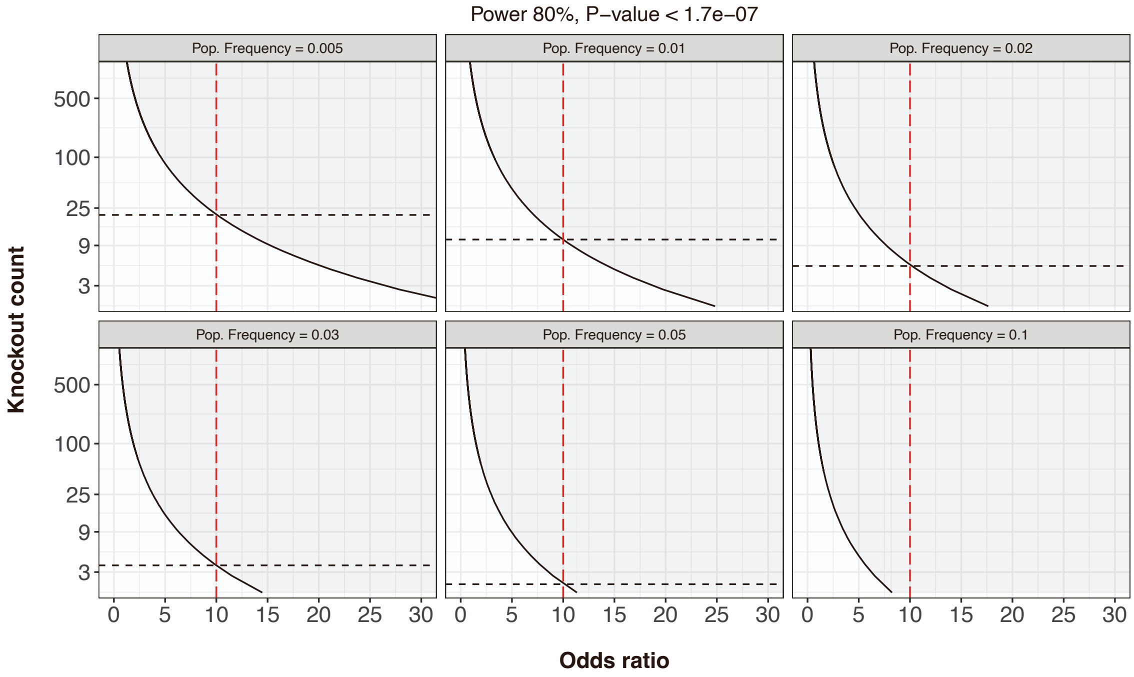

Figure S14: **Power analysis to determine the required number of bi-allelic variants to detect specific ORs at 80% power at bonferroni significance (**$P < 1.7 \times 10^{-7}$**), related to Star Methods.** We repeat the analysis while varying trait population prevalence assuming 823 (0.5%), 1766 (1%), 3532 (2%), 5298 (3%), 8829 (5%) cases out of 176,587 total individuals. The dashed red lines in the plot demonstrate the required number of bi-allelic variants to detect an OR $\geq$ 10.

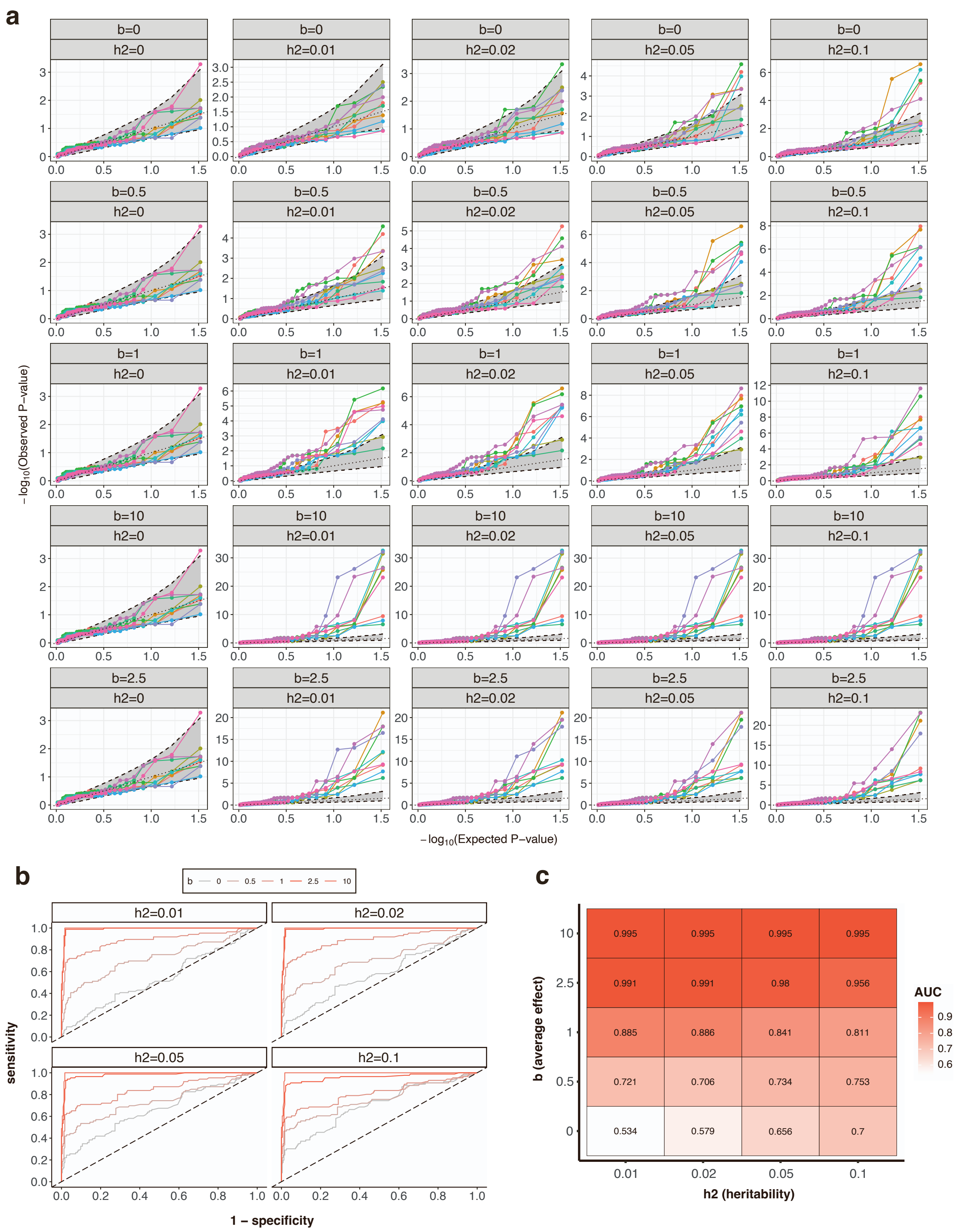
a
b=0
h2=0
h2=0.01
h2=0.02
h2=0.05
h2=0.1
b=0.5
b=1
b=10
b=2.5
$-\log_{10}$(Observed P−value)
$-\log_{10}$(Expected P−value)
b
b 0 0.5 1 2.5 10
h2=0.01
h2=0.02
h2=0.05
h2=0.1
sensitivity
1 − specificity
c
b (average effect)
h2 (heritability)
AUC
10: 0.995 0.995 0.995 0.995
2.5: 0.991 0.991 0.98 0.956
1: 0.885 0.886 0.841 0.811
0.5: 0.721 0.706 0.734 0.753
0: 0.534 0.579 0.656 0.7
0.01 0.02 0.05 0.1

Figure S15:

Figure S15 *(previous page)*: **Simulation study to test our ability to detect bi-allelic effects in the presence of true effects, related to Star Methods**. We simulate phenotypic data applied to 100,000 genetically-ascertained NFE on chromosome 22 (Methods) under the liability-threshold model assuming a spike and slab genetic architecture. We assume a 10% disease prevalence and 25% causal genes, and consider varying levels of phenotypic variance explained by these effects $\in \{0, 0.01, 0.02, 0.05, 0.10\}$. We then apply SAIGE to the simulated phenotypes, testing for an association between presence of a bi-allelic variant in each gene and case status. **a)** Each panel indicates a set of simulations assuming varying levels of heritability and average effect as labeled in the subtitles. In each panel, we plot the true effect size in the simulation for a given gene on x-axis against the corresponding $-\log_{10}(P)$ value of association. Areas of circles correspond to the number of samples harboring bi-allelic damaging variants in the 100,000 samples according to the legend. **b)** To assess the sensitivity and specificity of our approach, we created ROC-AUC curves for each combination of increasing phenotypic variance explained (facet) and increasing average affect (red lines). **c)** For each ROC-AUC curve from b, we calculate the AUC. White indicates low AUC and red indicates higher AUC.

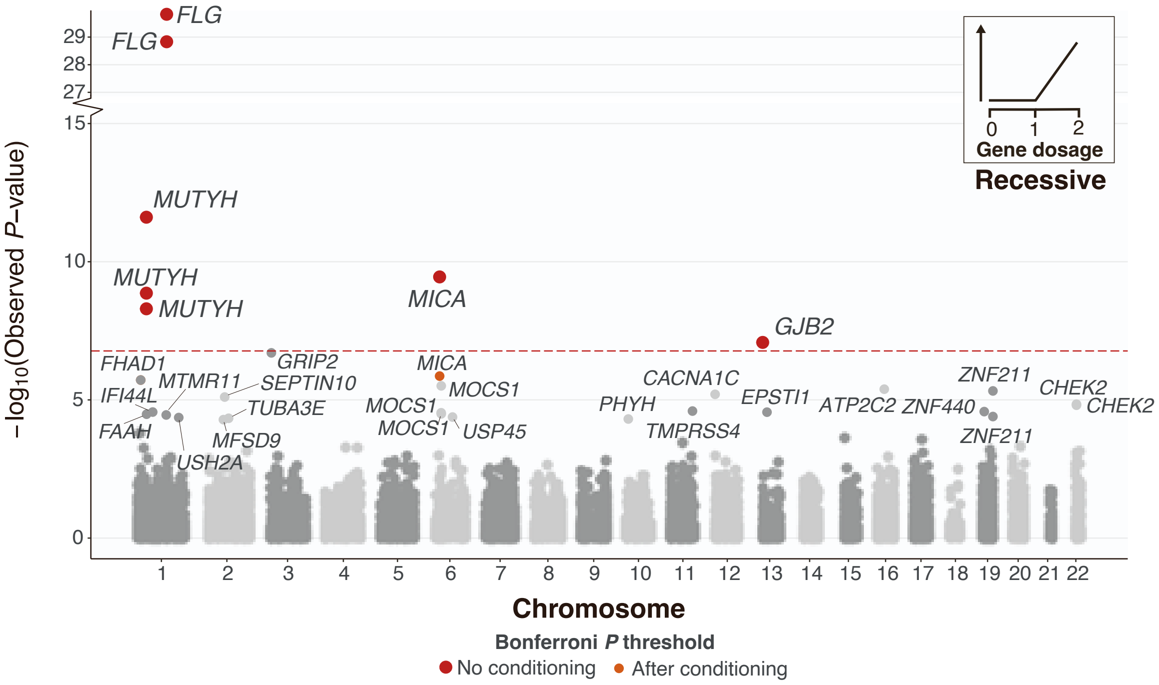

Figure S16: **Recessive association analysis without accounting for PRS, related to Figure 2**. Recessive Manhattan plot depicting $\log_{10}$-transformed gene-trait association P-values versus chromosomal location. Associations are colored red if they are Bonferroni significant ($P < 1.68 \times 10^{-7}$). Any gene-trait association with $P < 3.05 \times 10^{-6}$ (nominal significance) has been labeled with gene symbol. No additional conditioning was carried out in this analysis.

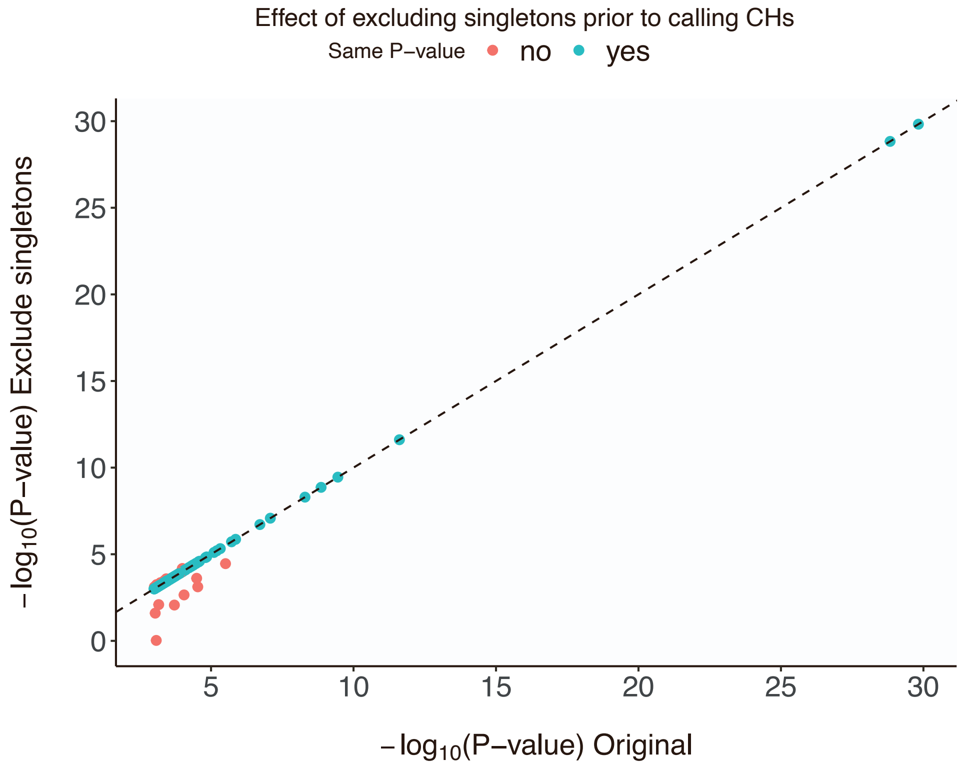

Figure S17: **Effect of excluding singletons prior to calling CH variants for any gene-traits with a** $P < 0.001$ **from the initial analysis, related to Figure 2**. The P-value from the original cross-sectional analysis is shown on the x-axis, while the P-value for the same associated gene-traits without singletons is depicted on the y-axis. The dots are colored by whether the degree of significance changes after excluding singletons.

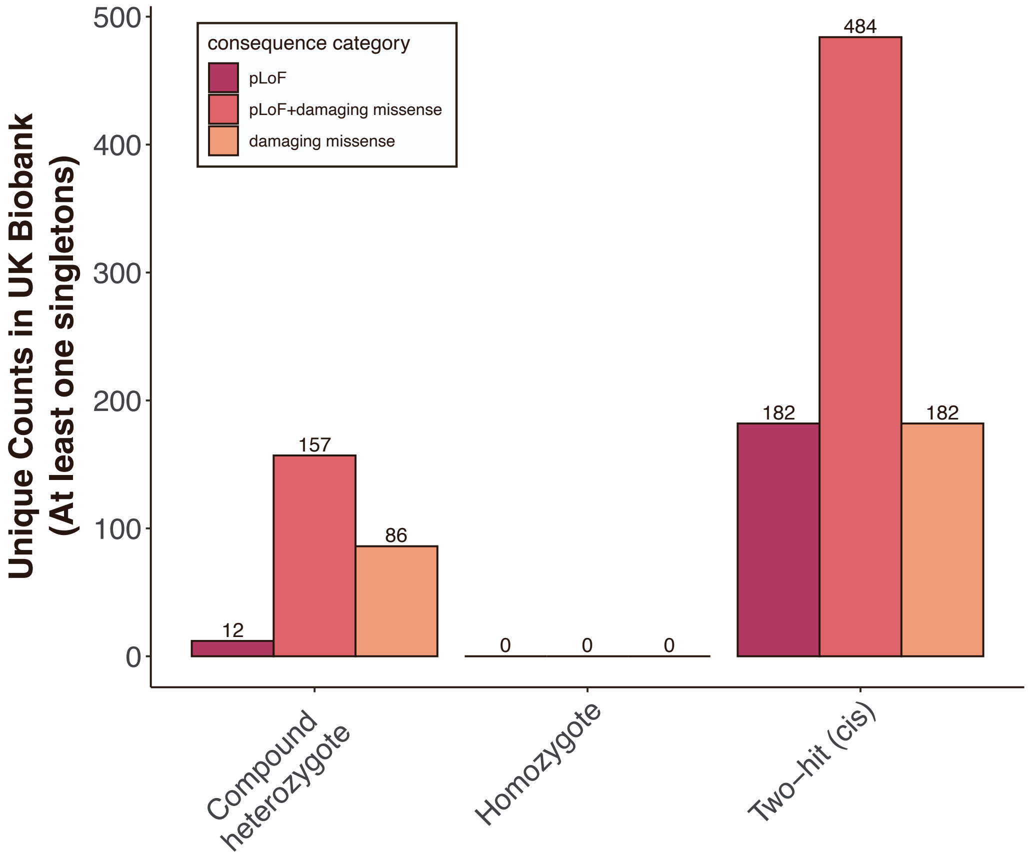

Figure S18: **Number of CH variants after filtering to hose that consist of at least one singleton, related to Figure 1**. We filter to CH variants that are comprised of at least one singleton across compound heterozygotes, homozygotes and carriers with variants *in cis*. The actual number of empirically observed carriers for each category is displayed on top of the each bar.

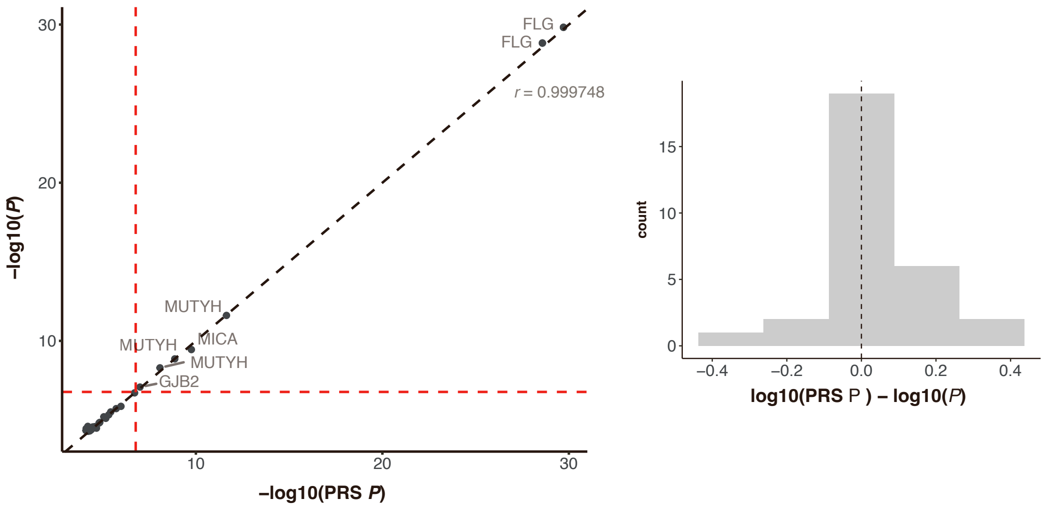

Figure S19: **Association *P*-values before and after inclusion of PRS as a covariate, related to Star Methods**. The scatter plot depicts the association P-values both before and after PRS was included as a covariate. The y-axis represents the P-value prior to PRS adjustment, while the x-axis demonstrates the P-value afterPRS adjustment. On the right, the difference in log-transformed P-values before and after PRS adjustment is displayed. The plot highlights gene-trait associations that were considered Bonferroni significant in the recessive analysis.

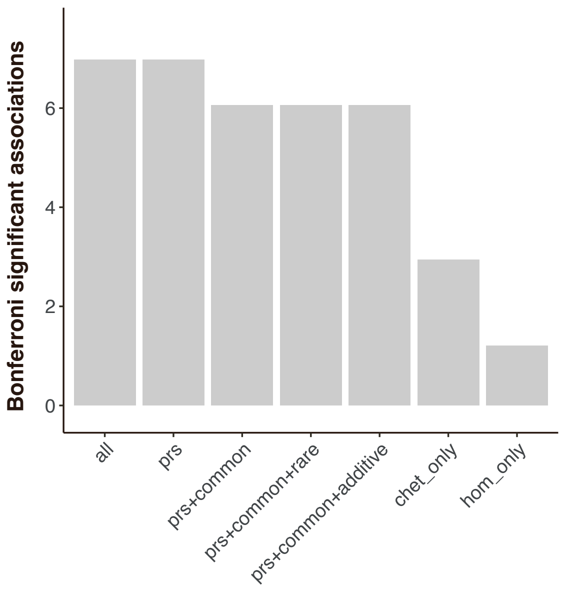

Figure S20: **Overview of attrition for Bonferroni significant associations after successive conditioning steps or filters, related to Star Methods**. This bar chart presents the number of Bonferroni significant associations that remain after successive conditioning steps in a gene-trait regression model or variant filters. The first bar represents all initial Bonferroni significant associations. The second bar shows the impact of conditioning on off-chromosome PRS. The third bar accounts for nearby common variants, which eliminates two gene-trait pairs. Subsequent bars indicate the effect of further conditioning on rare variants in the gene and an additive model of affected haplotypes, neither of which reduces the number of associations. The last two bars separate the associations those that remain after filtering to compound heterozygous or homozygous variants, respectively.

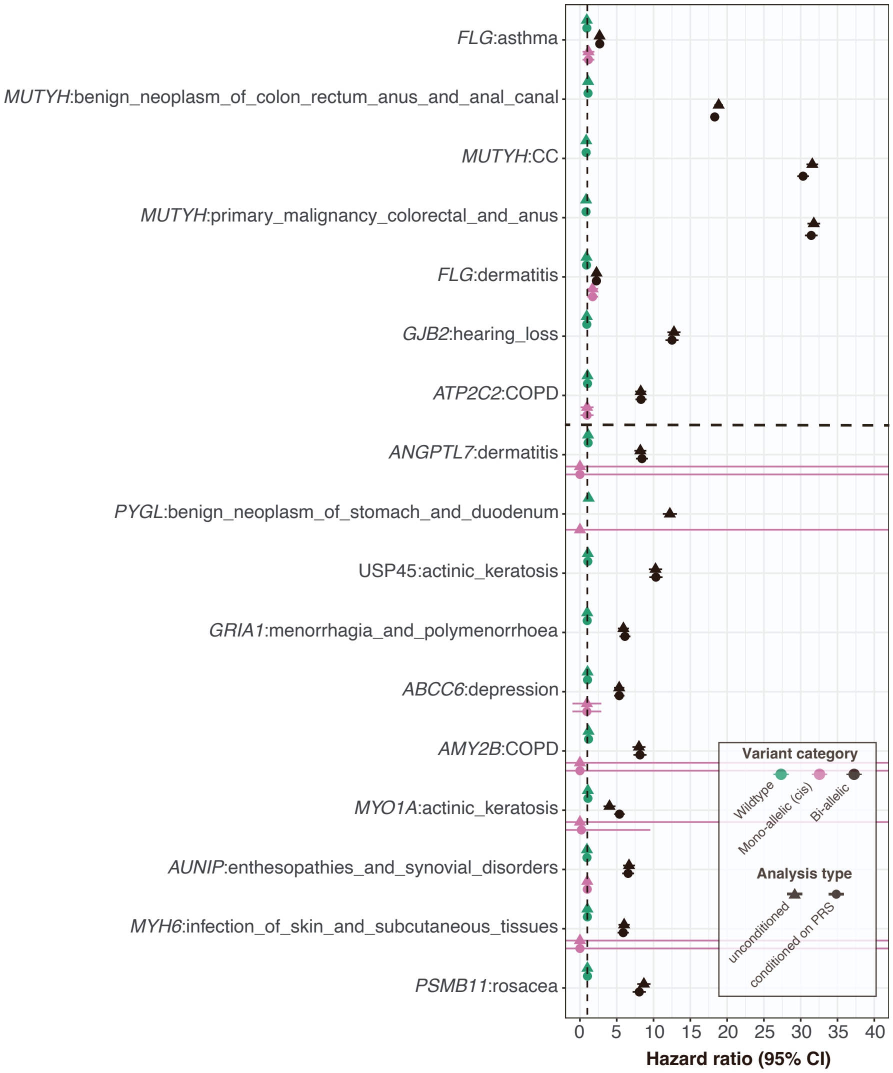

Figure S21: **Cox proportional hazards modeling with and without polygenic effects, related to Figure 4**. HRs when comparing CH and homozygous status versus heterozygous carrier status. Throughout, we display hazard ratios with (circles) and without (triangles) taking the polygenic contribution into account by conditioning on off-chromosome PRSs for heritable traits that pass our quality control cutoffs. HRs for gene-traits with one or more individuals with multiple *cis* variants on the same haplotype are also displayed in pink. Associations that pass either Bonferroni significance ($P < 1.89 \times 10^{-7}$) or FDRs < 0.1 cutoff are demarcated by the dashed line in the top and bottom half respectively. Abbreviations: CC (colorectal cancer), COPD (chronic obstructive pulmonary disease).

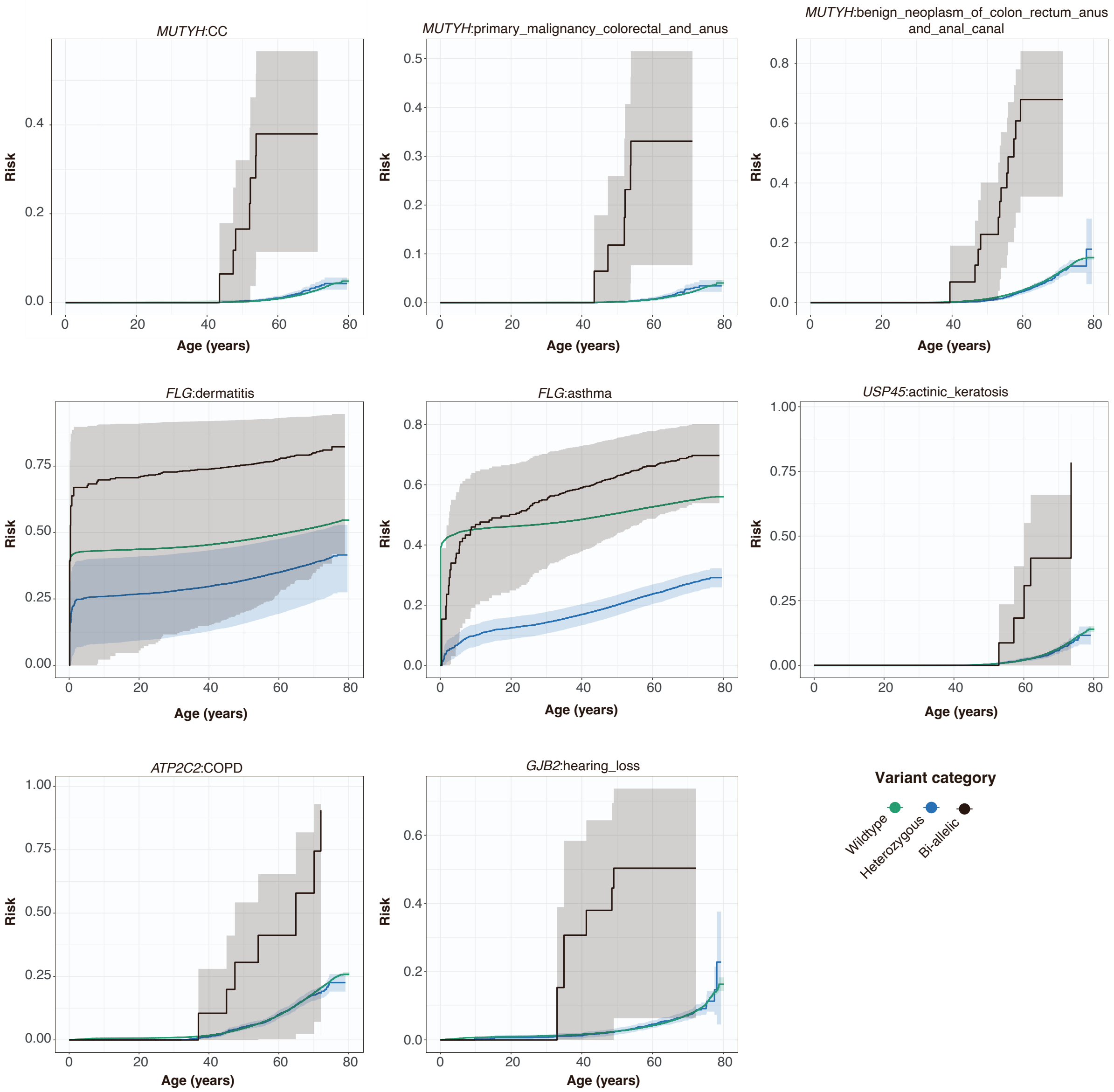

Figure S22: **Kaplan-Meier survival curves for carriers of bi-allelic variants, related to Figure 5**. Trajectories for wildtypes and bi-allelic (CH or homozygous) carriers of damaging missense/protein-altering mutations are shown with green and black lines respectively. For traits where over 50% of cases are left-censored, the confidence interval estimates cannot be accurately determined using Kaplan-Meier curves, and thus, these should be disregarded. Consequently, wildtype confidence intervals for *FLG*-Asthma and *FLG*-Dermatitis are not displayed in the figure.

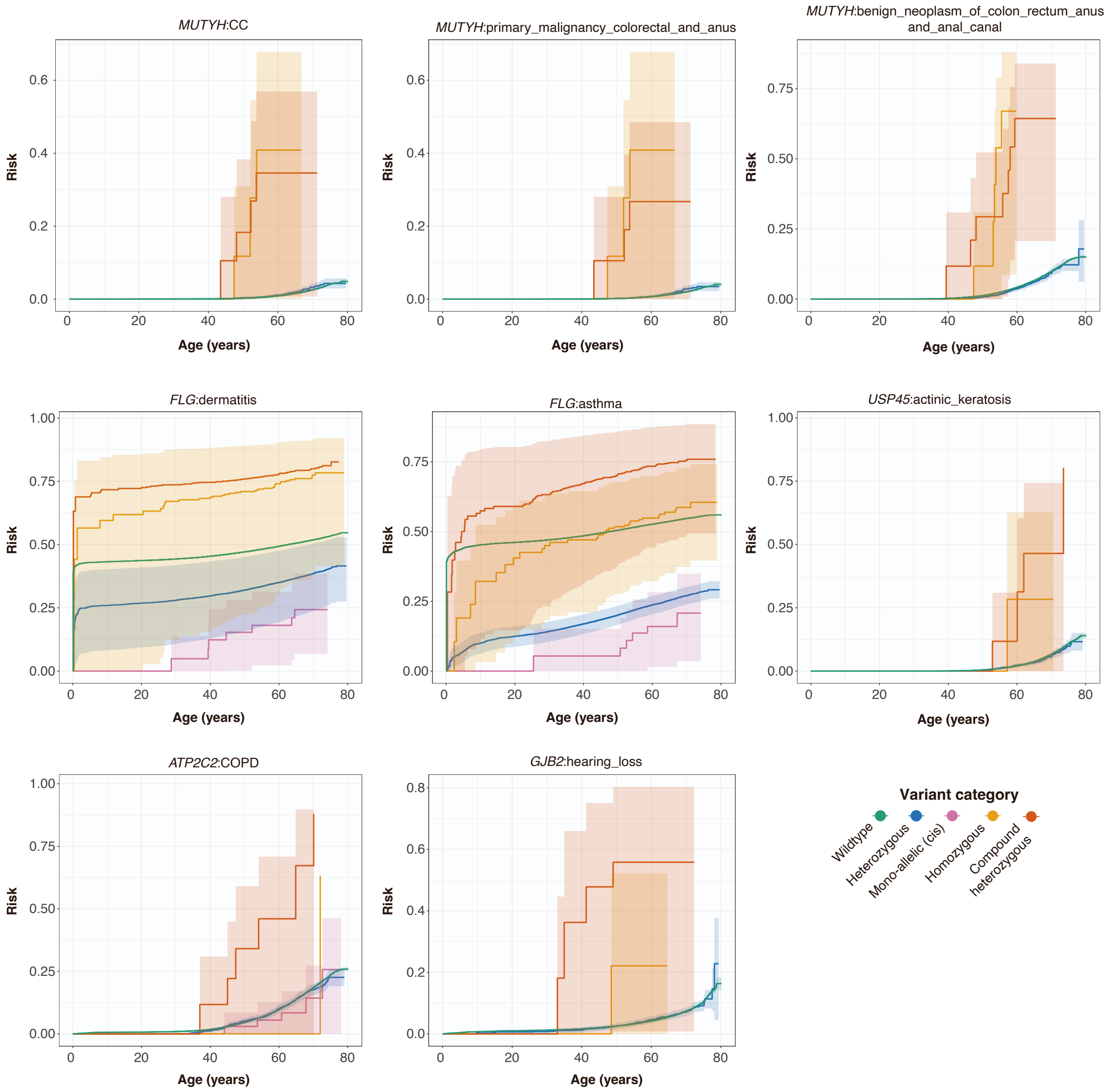

Figure S23: **Kaplan-Meier survival curves for carriers of CH, homozygous, heterozygous variants, related to Figure 5**. Kaplan-Meier survival curves for CH (red), homozygous (orange), heterozygous carriers (blue), single disruption of haplotypes (pink) owed to pLoF or damaging missense/protein-altering mutations. Wildtypes are shown in green. For traits where over 50% of cases are left-censored, the confidence interval estimates cannot be accurately determined using Kaplan-Meier curves, and thus, these should be disregarded. For this reason, wildtype confidence intervals for *FLG*-Asthma are not displayed in the figure. Wildtype and CH confidence intervals are also not shown for *FLG*-Dermatitis.

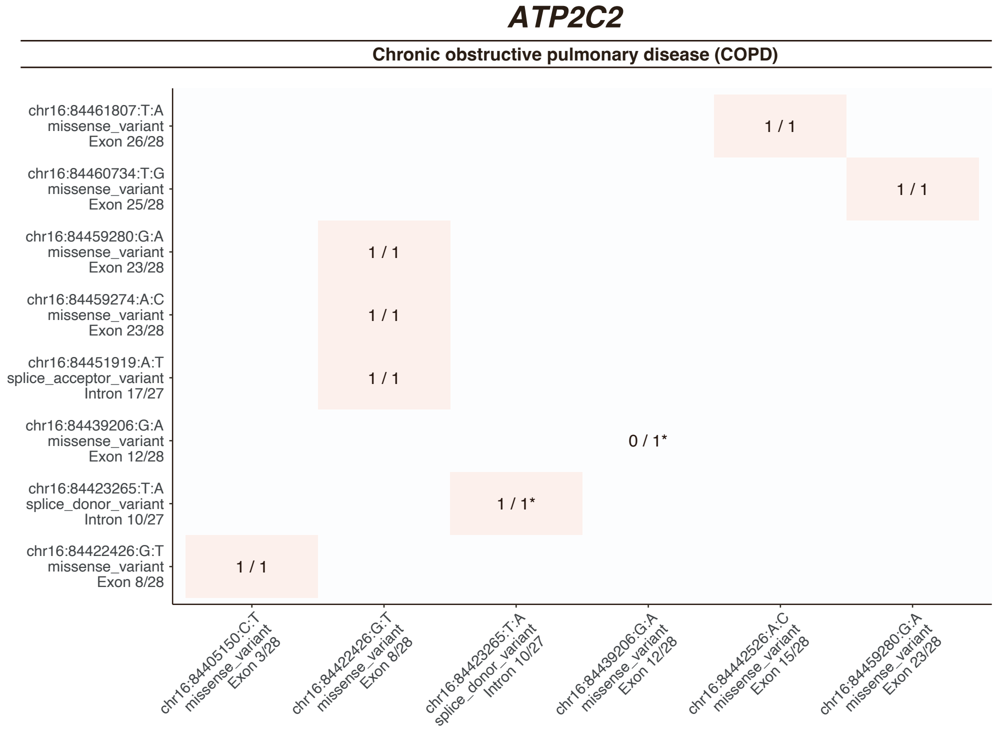

Figure S24: **Co-occurrence of deleterious *ATP2C2* variants by COPD status, related to Figure 5**. Bi-allelic variant occurrence in *ATP2C2* for chronic obstructive pulmonary disease (COPD). The constituent variants are shown alongside the variant consequence and involved exon or intron. Each tile indicates that number of individuals are cases out of the total bi-allelic carriers identified. Only the variants that affect both gene copies are shown. Stars (*) are included in the label to indicate homozygosity.

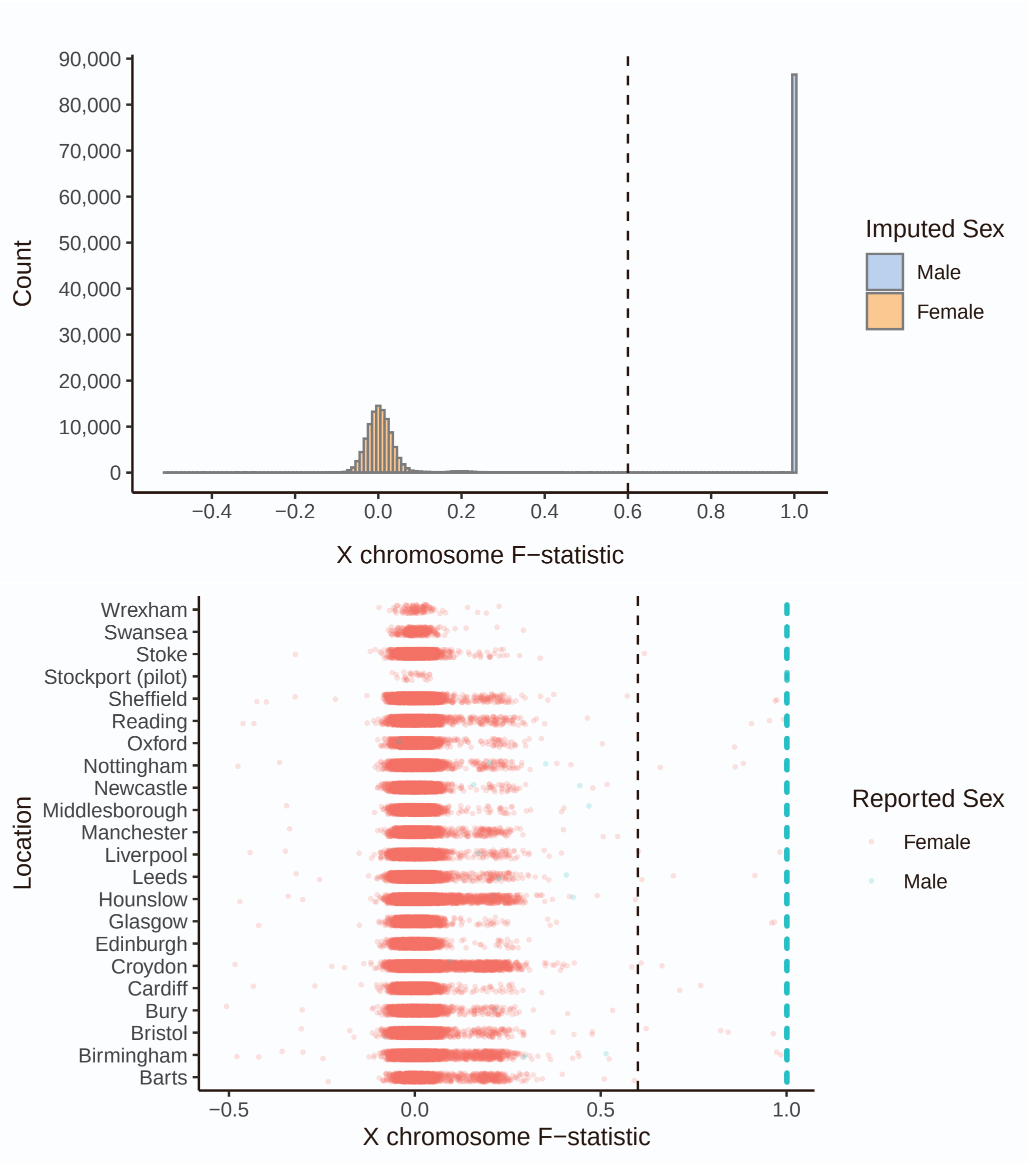

Figure S25: **Histogram and scatter-plots of X chromosome *F*-statistic by collection, related to Star Methods**. Samples lying to the left and right of the dashed line were called as female and male respectively, according to the imputed sex colorings in the upper histogram. Reported sex, split by UKBB recruitment center are shown in the lower jittered scatter-plots: red if the sample is reported as female, and blue if the sample is reported as male.

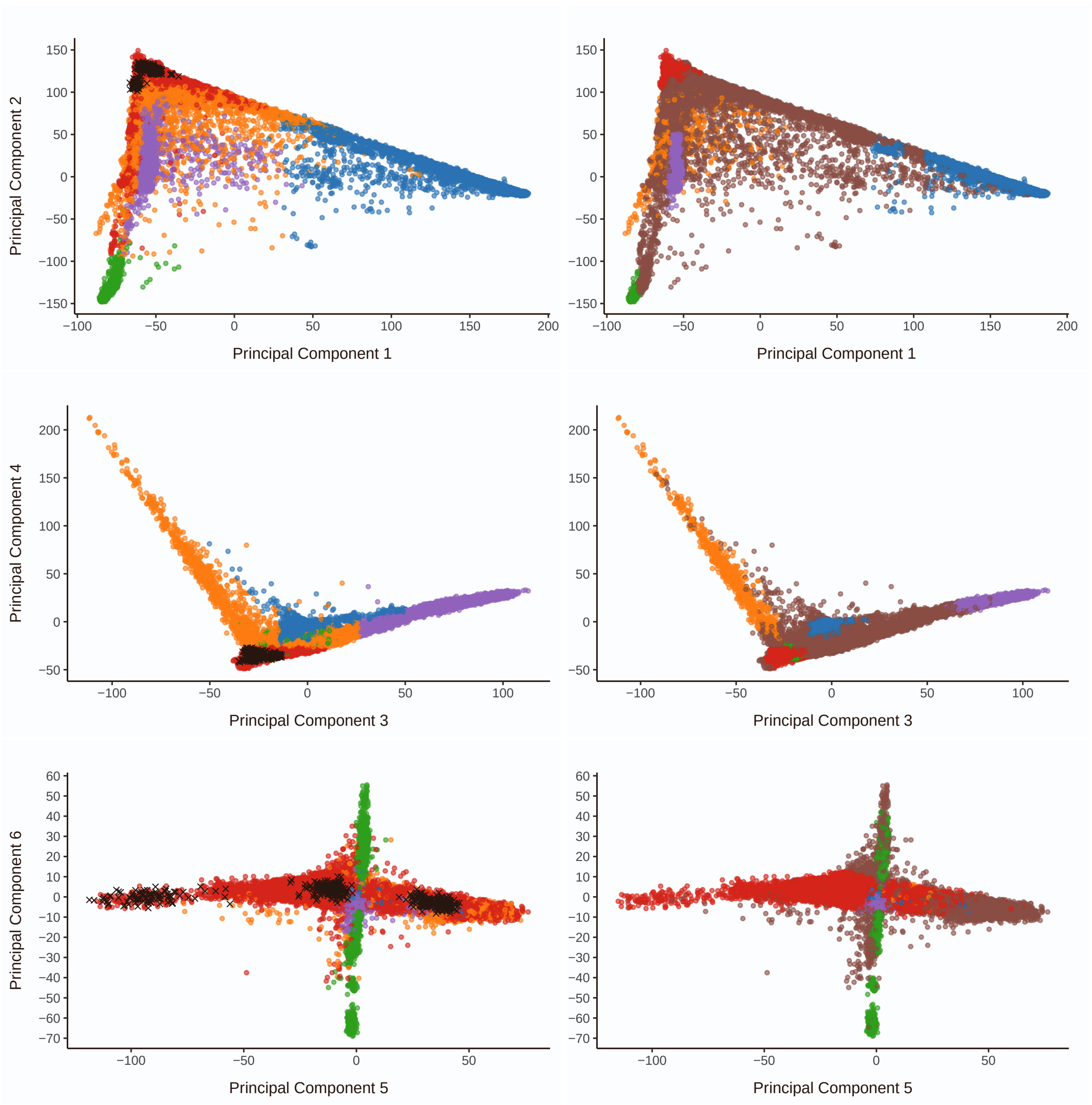

Figure S26: **Scatter-plots of PCs of UKBB genotype data projected into the PC space defined by 1KGP samples, related to Star Methods**. Points are colored according to sample collection, with 1KGP samples colored in blue. 1KGP super-populations labels were used to train a random forest classifier.

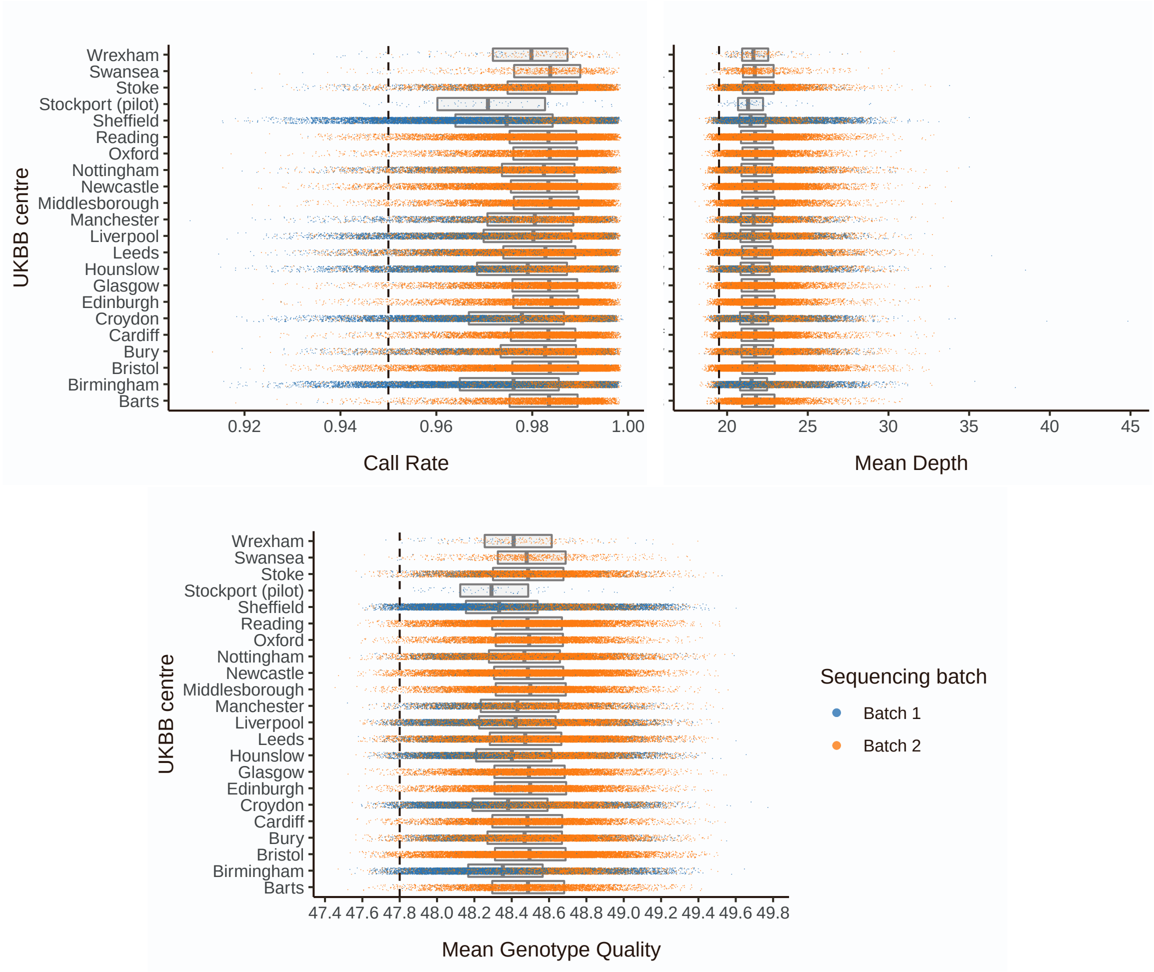

Figure S27: **Distributions of sample metrics following initial restriction to variants, lying outside LCRs and inside the padded (50 bp) target intervals, and prior to the initial hard sample filters (call rate > 0.95, mean depth > 19.5, mean GQ > 47.8), related to Star Methods**. In each plot, jittered scatters display the distribution for each UKBB recruitment center, colored according to sequencing batch. Box-plots behind the scatter display the median and interquartile range for each sequencing batch. Hard-filtering thresholds are denoted by the dashed vertical line.

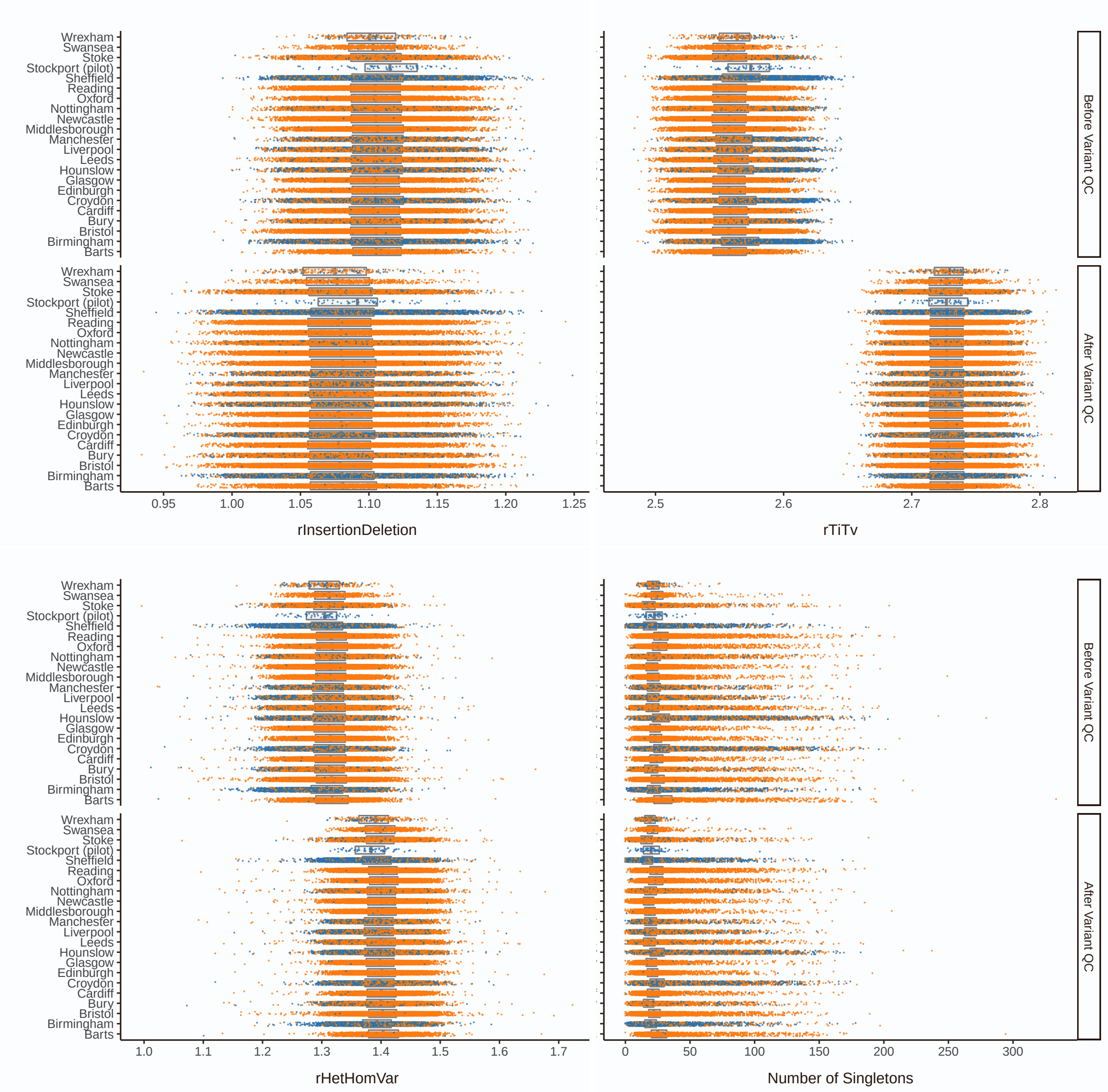

Figure S28: **Distributions of variant metrics before and after the removal of invariant sites, variants with call rate** < 0.97**, and variants out of HWE (**$P < 1 \times 10^{-6}$**), related to Star Methods**. In each plot, jittered scatters display the distribution for each sequencing batch colored by sequencing batch. Box-plots behind the scatter display the median and interquartile range for each sequencing batch. Points shown are following variants hard-filters and prior to removal of variants with metrics outside four standard deviations of the sequencing batch mean.
